# Stimuli-Responsive Rifampicin-Based Macromolecules

**DOI:** 10.3390/ma13173843

**Published:** 2020-08-31

**Authors:** Izabela Zaborniak, Angelika Macior, Paweł Chmielarz

**Affiliations:** 1 Department of Physical Chemistry, Faculty of Chemistry, Rzeszow University of Technology, al. Powstańców Warszawy 6, 35-959 Rzeszów, Poland; i.zaborniak@stud.prz.edu.pl; 2 School of Engineering and Technical Sciences, Rzeszow University of Technology, al. Powstańców Warszawy 8, 35-959 Rzeszów, Poland; angelika.macior@onet.pl

**Keywords:** rifampicin-based ATRP initiator, preparative electrolysis, stimuli-responsive polymer materials

## Abstract

This paper presents the modification of the antibiotic rifampicin by an anionic polyelectrolyte using a simplified electrochemically mediated atom transfer radical polymerization (*se*ATRP) technique to receive stimuli-responsive polymer materials. Initially, a supramolecular ATRP initiator was prepared by an esterification reaction of rifampicin hydroxyl groups with α-bromoisobutyryl bromide (BriBBr). The structure of the initiator was successfully proved by nuclear magnetic resonance (^1^H and ^13^C NMR), Fourier-transform infrared (FT-IR) and ultraviolet–visible (UV-vis) spectroscopy. The prepared rifampicin-based macroinitiator was electrochemically investigated among various ATRP catalytic complexes, by a series of cyclic voltammetry (CV) measurements, determining the rate constants of electrochemical catalytic (EC’) process. Macromolecules with rifampicin core and hydrophobic poly (*n*-butyl acrylate) (P*n*BA) and poly(*tert*-butyl acrylate) (P*t*BA) side chains were synthesized in a controlled manner, receiving polymers with narrow molecular weight distribution (*M*_w_/*M*_n_ = 1.29 and 1.58, respectively). “Smart” polymer materials sensitive to pH changes were provided by transformation of *t*BA into acrylic acid (AA) moieties in a facile route by acidic hydrolysis. The pH-dependent behavior of prepared macromolecules was investigated by dynamic light scattering (DLS) determining a hydrodynamic radius of polymers upon pH changes, followed by a control release of quercetin as a model active substance upon pH changes.

## 1. Introduction

Polymers as multifunctional structures are intensively studied in drug formulation and drug delivery systems [1,2,3,4]. It is directly connected with unique properties of polymers to respond to external stimuli, i.e., thermoresponsive macromolecules, such as a widely known poly(*N*-isopropylacrylamide) [5] or recently studied poly(2-isopropenyl-2-oxazoline) [6], and polyelectrolytes with positively or negatively charged groups, sensitive to pH changes, e.g., anionic poly(acrylic acid) (PAA) [7] or cationic poly(2-(dimethylamino)ethyl methacrylate) [8], making them “smart” materials and promising candidates to control encapsulate and release of drugs, and cell-specific targeting [1,9,10]. There is a wide range of nontoxic, biocompatible, and biodegradable polymeric materials, successfully implemented in the controlled release of different substances upon pH changes. This subject is especially privileged among antibiotics delivery systems. Polymers are usually used as separate structures, that encapsulate and release drugs upon external stimulus changes [8,10,11]. In this work, the incorporation of polymer chains into a rifampicin structure is proposed to incorporate polymer chains into the structure of rifampicin. Direct modification of drugs by an appropriate polymer provides additional benefits beyond the possibility for extended-release delivery systems, namely, improves the property of drugs [12] and can avoid the resistance of antibiotics for bacteria [13].

Rifampicin is a powerful heterocyclic antibiotic, used as a first-line drug for the treatment of tuberculosis, due to easy diffusion into tissue, living cells, and bacteria [14,15]. Rifampicin is classified to ansamycins because of its heterocyclic structure consisting of an aromatic chromophore (naphthoquinone core) that is responsible for its characteristic red-orange crystalline color, spanned by an aliphatic ansa chain [16]. Due to the presence of hydroxyl groups in its structure, it is an excellent candidate for modification by atom transfer radical polymerization (ATRP) methods [17,18,19,20]. ATRP belongs to the reversible deactivation radical polymerization (RDRP) technique, based on the use of a transition metal complex as a catalyst that participates in redox reactions and thus controls polymerization [21,22,23]. Among other RDRP techniques, it is recently the most influential approach in the preparation of precisely-defined polymer materials with complex architecture [24,25]. It was successfully applied for the synthesis of a wide range of branched architectures by modification of naturally-derived and bioactive structures, e.g., vitamins [17,26,27], sugars [28,29], tannins [30,31], drugs [32], and biopolymers [33,34,35], according to the “grafting from” approach, providing materials with predetermined structure for potential use in biomedical applications. The use of ATRP for drug modification is beneficial from several reasons, i.e., control of the polymer chain structure during polymerization resulting in materials with precise functionality, ability to receive ultra-pure products using “clean” techniques controlled by external stimulus e.g., electric current [36,37,38], ultrasound [39,40,41] and light [42,43,44,45], removing additional chemical reducing agents from reaction media; externally control methods allow for temporal control during polymerization, beneficial in the preparation of predetermined structures [17,27,36]; low ppm ATRP approaches use only ppm level of catalyst, easily removed from final products, without contamination of the product [46,47]. Up to now, electrochemically-mediated ATRP (*e*ATRP) is the most controlled technique among externally controlled ATRP methods. The precise control of the ratio between Cu^I^ and Cu^II^ during polymerization is established by constant current (*I*) or potential (*E*) at the working electrode surface [17,48,49,50]. It provides precisely-defined branched polymeric materials by avoiding an undesirable termination reaction and gelation with the increase of molecular weight [51,52,53]. Reaction setup in preparative electrolysis consists of a three-electrode system. It can be simplified by replacing a platinum electrode constituting a counter electrode by an aluminum immersed directly in reaction media, making this technique simplified electrochemically-mediated ATRP (*se*ATRP) [36,54]. Another simplification is an application of constant current (galvanostatic) conditions, that removes a reference electrode from the electrochemical cell [27,55]. Additionally, *se*ATRP as an external control approach was successfully temporally controlled in the preparation of both well-defined linear and branched architectures [18,27,56].

In this study, the synthetic route for the modification of rifampicin using *se*ATRP method was investigated. The first step of the “grafting from” approach covers the preparation of rifampicin-based multifunctional ATRP macroinitiator by esterification of rifampicin with α-bromoisobutyryl bromide (BriBBr). The prepared supramolecular structure was electrochemically investigated for the use as an ATRP initiator in the presence of different catalyst complexes, determining rate constant of the electrochemical catalytic process (*k*_EC’_)—characteristics of ATRP polymerization. The polymer brushes with rifampicin core and hydrophobic poly(*n*-butyl acrylate) (P*n*BA) and poly(*tert*-butyl acrylate) (P*t*BA) side chains were synthesized. pH-responsive “smart” rifampicin-based materials were received by the transformation of *t*BA units into acrylic acid (AA) moieties. PAA has anionic functional groups that become ionized in response to pH changes, resulting in control release properties.

## 2. Materials and Methods

### 2.1. Chemicals

Rifampicin (Rif, *M*_n_ = 922.94, 95%, Acros Organics, Morris Plains, NJ, USA), 2-bromoisobutyryl bromide (BriBBr, 98%, Sigma-Aldrich, St. Louis, MO, USA), *N*-methyl-2-pyrrolidone (NMP, >99%, Sigma-Aldrich), dichloromethane (DCM, >99.9%, Sigma-Aldrich), tetrabutylammonium perchlorate (TBAP, >98%, Sigma-Aldrich), *N*,*N*-dimethylformamide (DMF, >99.9%, Honeywell Riedel-de Haen, Muskegon, MI, USA), copper(II) bromide (Cu^II^Br_2_, 99.9%, Sigma-Aldrich), *N,N,N’,N”,N”*-pentamethyldiethylenetriamine (PMDETA, >98%, Sigma-Aldrich), 2,2,6,6-tetramethylpiperidine 1-oxyl (TEMPO, 98%, Sigma-Aldrich), methanol (MeOH, >99.8%, Sigma-Aldrich), water (ACS Reagent, Honeywell Riedel-de Haen), trifluoroacetic acid (TFA, >99%, Sigma-Aldrich), tetrahydrofuran (THF, >99.9%, Sigma Aldrich), *n*-butanol (>99.7%, Sigma-Aldrich), sulfuric acid (>95%, Sigma-Aldrich), sodium bicarbonate (NaHCO_3_, >99.7%, Sigma-Aldrich), diiodomethane (99%, Sigma-Aldrich), sodium hydroxide (NaOH, >98%, Honeywell Riedel-de Haen), hydrochloric acid (HCl, 35–38%, Chempur, Piekary Śląskie, Poland), and buffer solutions (pH 1 and pH 9, POCH, Gliwice, Poland) were not subjected to further purification. Tris (2-pyridylmethyl)amine (TPMA), bis (4-methoxy-3, 5-dimethyl-pyridin-2-ylmethyl)-pyridin-2-ylmethyl-amine (TPMA*^2^) [57,58], and Cu^II^Br_2_/TPMA catalyst complex were prepared as previously reported [26,59]. *n*-Butyl acrylate (BA, >99% Sigma-Aldrich) and *tert*-butyl acrylate (*t*BA, >99%, Sigma-Aldrich) were passed through a column filled with basic alumina before use in order to remove inhibitor. Pt gauze (99.9% metals basis), Pt mesh, Al wire, and Pt disk (3 mm diameter, Gamry Instruments, Warminster, PA, USA) were purchased from Alfa Aesar (Ward Hill, MA, USA).

### 2.2. Analysis

Subsequently, ^1^H and ^13^C NMR analysis was carried out in CDCl_3_ using Bruker Avance 500 MHz spectrometer (Bruker, Karlsruhe, Germany) in 25 °C. Gel permeation chromatography (GPC) was performed to determine Molecular weights (MWs) and molecular weight distributions (MWD, *M*_w_/*M*_n_, *Ð*) using Viscotek, T60A detector (Houston, TX, USA) equipped with polymer standards services (PSS) columns (guard 10^3^ and 10^2^ Å). The eluent was HPLC grade THF, and the flow rate was 1 mL/min. Calibration curve was generated on the basis of monodispersed polystyrene (PS) standards using TRISEC software from Viscotec Corporation (version 3.0). Volume mean diameter (*d*_volume_) of polymer sample (1 mg/mL in THF) was measured by Dynamic Light Scattering (DLS, Zetasizer Nano ZS, Malvern Panalytical, Worcestershire, UK) at 20 °C. FT-IR spectra were taken with the spectrophotometer Nicolet 6700 FT-IR (Thermo Scientific, Waltham, MA, USA), within 500–4000 cm^–1^, with the use of attenuated total reflectance (ATR) technique. The UV–vis spectra were obtained on a Hewlett-Packard (Waldbronn, Germany) Model HP-8453 diode array rapid scan spectrophotometer using a quartz cell with optical length of 1 cm. Cyclic voltammetry (CV) measurements and preparative electrolysis were performed using an Autolab model AUT84337 potentiostat (Metrohm, Utrecht, The Netherlands) running with a GPES software in a five-neck electrochemical cell equipped electrodes as follows: working electrode (WE)—Pt disk (A = 0.071 cm^2^, carefully polished with 0.05 μm alumina suspension (Buehler) before every single measurement) for CV and Pt mesh for preparative electrolysis (A = ~6 cm^2^), reference electrode (RE)—saturated calomel electrode (SCE), equipped with a saturated salt bridge and a Vycor tip, immersed inside a Luggin capillary, counter electrode (CE)—an Al wire (*l* = 10 cm, *d* = 1 mm) immersed directly in the reaction mixture. During preparative electrolysis a condenser was connected to the reaction cell kit and the temperature was maintained at 50 °C (thermostat ESM-3711-H, Laboplay, Bytom, Poland).

### 2.3. Synthesis of Rifampicin-Based ATRP Macroinitiator (Rif-Br_3_)

Rifampicin (0.8 g, 0.97 mmol) was dissolved in NMP (20 mL) under Ar atmosphere in a 100 mL round bottom flask. A solution of BriBBr (2.40 mL, 19.4 mmol) in NMP (5.6 mL) was added dropwise over a period of 1 h at 0 °C (ice bath). Then the solution was removed from the ice bath and stirred in a sealed flask for 7 days at room temperature. Upon completion, the reaction mixture was diluted with DCM (30 mL), washed with water (100 mL × 15), and the organic phase was placed in dialysis tubing (Spectra/Por dialysis membrane, MWCO 1000) and dialyzed against deionized water for 10 cycles over 7 days. Subsequently, the product concentrated under reduced pressure. The resulting dark red solid product was dried under vacuum (0.81 g, yield 66%). The final ATRP macroinitiator was characterized by NMR, GPC, FT-IR, and UV-vis analysis (Supplementary Figures S1–S4, respectively).

### 2.4. Electrochemical Characterization

Electrochemical characterization of Rif-Br_3_ in the presence of three different catalytic complexes (Cu^II^Br_2_/PMDETA, Cu^II^Br_2_/TPMA, Cu^II^Br_2_/TPMA*^2^) was performed. The Pt disc, Al wire, and SCE were prepared and located in the electrochemical cell. Rif-Br_3_, Cu^II^Br_2_/L (0.05 M in DMF) and TEMPO were dissolved in DMF in the ratio [Rif-Br_3_]/[Cu^II^Br_2_/L]/[TEMPO] = 1:0.08:4. The mixture was Ar purged. The first series of CV measurements in different scan rates were carried out after addition of catalytic complex. The next series of CV measurements were conducted after the Rif-Br_3_ and TEMPO were introduced to the electrochemical cell.

### 2.5. The Synthesis of Rif-(PnBA-Br)_3_ by seATRP under Constant Potential Conditions with Cu^II^Br_2_/TPMA*^2^ Catalytic Complex

The Pt disc, Al wire, and SCE were prepared and located in the five-neck jacked electrochemical cell equipped with condenser. 1.71 g of TBAP (5.0 mmol), 3.8 mL of BA (27.4 mmol), 20.0 mL of DMF and 219 μl of Cu^II^Br_2_/TPMA*^2^ stock solution (0.05 M in DMF) were introduced into the cell at 50 °C under a slow Ar purge. CV of catalytic complex was recorded to determine the appropriate applied potential (*E*_app_ = −0.400 V; Supplementary Figure S8a) during *se*ATRP. Then, 43.0 mg of Rif-Br_3_ (33.9 µmol) in 1 mL of DMF was added and the CV of catalytic complex in the presence of ATRP initiator was recorded. The Pt disc was replaced by Pt mesh and after degassing the preparative electrolysis was conducted. Samples were withdrawn periodically to follow the monomer conversion by ^1^H NMR, and determine *M*_n_ and *M*_w_/*M*_n_ by GPC. Before GPC analysis, the sample was dissolved in methanol, precipitated in water, dried under vacuum for 1 day, dissolved in THF, passed through a neutral alumina column with 0.22 µm filter.

### 2.6. The Synthesis of Rif-(PnBA-Br)_3_ by seATRP under Constant Potential Conditions with Cu^II^Br_2_/TPMA Catalytic Complex

The Pt disc, Al wire, and SCE were prepared and located in the five-neck jacked electrochemical cell equipped with condenser. 1.09 g of TBAP (3.2 mmol), 4.8 mL of *n*BA (35.1 mmol), 9.92 mL of DMF and 281 μL of Cu^II^Br_2_/TPMA stock solution (0.05 M in DMF*)* were introduced into the cell at 50 °C under a slow Ar purge. CV of catalytic complex was recorded to determine the appropriate applied potential (*E*_app_ = −0.320 V; Supplementary Figure S9a) during *se*ATRP. Then, 55 mg of Rif-Br_3_ (43.3 µmol) in 1 mL of DMF was added and the CV of catalytic complex in the presence of ATRP initiator was recorded. The Pt disc was replaced by Pt mesh and after degassing the preparative electrolysis was conducted. Samples were withdrawn periodically to follow the monomer conversion by ^1^H NMR, and determine *M*_n_ and *M*_w_/*M*_n_ by GPC. Before GPC analysis, the sample was dissolved in methanol, precipitated in water, dried under vacuum for 1 day, dissolved in THF, and passed through a neutral alumina column with a 0.22 µm filter.

### 2.7. The Synthesis of Rif-(PnBA-Br)_3_ by Temporally-Controlled seATRP under Constant Current Conditions with Cu^II^Br_2_/TPMA Catalytic Complex

The Pt mesh and Al wire were prepared and located in the five-neck jacked electrochemical cell equipped with condenser. 1.09 g of TBAP (3.2 mmol), 4.8 mL of *n*BA (35.1 mmol), 9.92 mL of DMF, 281 μL of Cu^II^Br_2_/TPMA stock solution (0.05 M in DMF) and 55 mg of Rif-Br_3_ (43.3 µmol) in 1 mL of DMF were introduced into the cell at 50 °C under a slow Ar purge. After degassing the preparative electrolysis was conducted. Samples were withdrawn periodically to follow the monomer conversion by ^1^H NMR, and determine *M*_n_ and *M*_w_/*M*_n_ by GPC. Before GPC analysis, the sample was dissolved in methanol, precipitated in water, dried under vacuum for 1 day, dissolved in THF, passed through a neutral alumina column with a 0.22 µm filter. The final purified polymer product was characterized by ^1^H NMR (Supplementary Figure S13).

### 2.8. The Synthesis of Rif-(PtBA-Br)_3_ by seATRP under Constant Potential Conditions with Cu^II^Br_2_/TPMA Catalytic Complex

The Pt disc, Al wire, and SCE were prepared and located in the five-neck jacked electrochemical cell equipped with condenser. Then, 1.09 g of TBAP (3.2 mmol), 4.8 mL of *t*BA (35.1 mmol), 9.94 mL of DMF, and 262 μL of Cu^II^Br_2_/TPMA stock solution (0.05 M in DMF) were introduced into the cell at 50 °C under a slow Ar purge. CV of catalytic complex was recorded to determine the appropriate applied potential (*E*_app_ = −0.322 V; Supplementary Figure S11a) during *se*ATRP. Then, 137 mg of Rif-Br_3_ (0.11 mmol) in 1 mL of DMF was added and the CV of catalytic complex in the presence of ATRP initiator was recorded. The Pt disc was replaced by Pt mesh and after degassing the preparative electrolysis was conducted. Samples were withdrawn periodically to follow the monomer conversion by ^1^H NMR, and determine *M*_n_ and *M*_w_/*M*_n_ by GPC. Before GPC analysis, the sample was dissolved in methanol, precipitated in water, dried under vacuum for 1 day, dissolved in THF, passed through a neutral alumina column with a 0.22 µm filter. The final purified polymer product was characterized by ^1^H NMR (Supplementary Figure S14).

### 2.9. The Synthesis of Rif-(PtBA-b-PtBA-Br)_3_ by seATRP under Constant Potential Conditions with Cu^II^Br_2_/TPMA Catalytic Complex

The Pt disc, Al wire, and SCE were prepared and located in the five-neck jacked electrochemical cell equipped with condenser. 0.96 g of TBAP (2.8 mmol), 2.2 mL of *t*BA (14.9 mmol), 7.69 mL of DMF. and 119 μl of Cu^II^Br_2_/TPMA stock solution (0.05 M in DMF) were introduced into the cell at 50 °C under a slow Ar purge. CV of catalytic complex was recorded to determine the appropriate applied potential (*E*_app_ = −0.320 V; Supplementary Figure S12a) during *se*ATRP. Then, 844 mg of Rif-(P*t*BA-Br)_3_ (22.1 µmol) in 4 mL of DMF was added and the CV of catalytic complex in the presence of ATRP initiator was recorded. The Pt disc was replaced by Pt mesh and after degassing the preparative electrolysis was conducted. Samples were withdrawn periodically to follow the monomer conversion by ^1^H NMR, and determine *M*_n_ and *M*_w_/*M*_n_ by GPC. Before GPC analysis, the sample was dissolved in methanol, precipitated in water, dried under vacuum for 1 day, dissolved in THF, and passed through a neutral alumina column with a 0.22 µm filter. The final purified polymer product was characterized by ^1^H NMR (Supplementary Figure S15).

### 2.10. Detaching Polymer Side Chains from Rifampicin-Based Macromolecules

The detachment of the polyacrylates side chains was conducted by acid solvolysis according to the previously described procedure [60].

### 2.11. Transformation of PtBA to PAA Side Chains

The P*t*BA blocks of rifampicin-based macromolecules were hydrolysed to PAA blocks (Supplementary Figure S7). The Rif-(P*t*BA-Br)_3_ (0.17 g, 4.5 µmol) was dissolved in DCM (10 mL) and a 5-fold molar excess of TFA with respect to the ester groups (0.4 mL, 5.2 mmol) was added. The Rif-(P*t*BA-*b*-P*t*BA-Br)_3_ (0.42 g, 5.9 µmol) was dissolved in DCM (15 mL) and a 5-fold molar excess of TFA with respect to the ester groups (0.7 mL, 9.4 mmol) was added. The mixtures was stirred at room temperature for 72 h. When P*t*BA moieties were hydrolyzed, the PAA-based macromolecules precipitate in DCM. They were separated by filtration, washed with DCM, and the solvent and TFA were removed by rotating evaporation. The final sample was dried under vacuum at 50 °C for 1 day and characterized by ^1^H NMR (Supplementary Figures S17 and S18), and FT-IR analysis (Supplementary Figures S19 and S20).

### 2.12. Confirmation of PtBA Hydrolysis to PAA Moieties by Contact Angle Measurements

The character of the wetting PAA-based and corresponding P*t*BA-based polymer film was measured by the determination of contact angles values (θ). Silicon wafers were coated by the solution of a polymer in water (PAA, 5 mg/mL) and THF (P*t*BA, 5 mg/mL). After evaporation of solvents, (10 µL) of water (polar) and diiodomethane (non-polar) liquid was put on the prepared polymer films. Pictures of the droplets were taken (Camera 13 MP, f/2.2, PDAF) each time three droplets of the individual liquids were analyzed. The geometric analysis of pictures was conducted by *Kropla* software, receiving the values of contact angles, followed by the calculation of free surface energy (FSE, γ_S_) values for polymer coatings according to Owens–Wendt methods by software *Energia* [31].

### 2.13. Determination of pH-Sensitivity of PAA-Based Polymers

The behavior of the rifampicin-based macromolecules with PAA side chains against pH changes was investigated by potentiometric titration, determining hydrodynamic radius of polymers by DLS measurements of the solutions in different pH values. Potentiometric titration was performed using a digital pH meter (CPC-551, ELMETRON, Zabrze, Poland), equipped with a combined glass/reference electrode (HYDROMET, ERH-13-6). The polymer was dissolved in 0.1 M NaOH (1 mg/mL solution). pH value was adjusted by titration with aqueous 0.1 M HCl under intensive stirring, at a constant temperature (25 °C). pH readings were registered after each portion of titrant added to polymer solution, when the equilibrium state was established.

### 2.14. Loading of Quercetin (QC) into the PAA-Based Polymer and Its Release Behavior upon pH Changes

Quercetin loaded micelles were prepared by the dialysis method as fallow. Initially, 20 mg of Rif-(P*t*BA-*b*-P*t*BA-Br)_3_ polymer and 8 mg of QC was dissolved in DMF, and stirred for half an hour. The mixed solution was added dropwise into 20 mL water, stirred for 1.5 h, placed in dialysis tubing (Spectra/Por dialysis membrane, MWCO 1000) and dialyzed against deionized water for 2 cycles over 16 hours. The received QC loaded micelles solution was adjusted to 70 mL by DMF, and the total mass of QC loaded in PAA-based micelles was estimated on the basis of calibration curve of the absorption intensity at λ = 374 nm as a function of QC concentration in DMF. The loading efficiency (*E*_L_) was calculated as follows [61]:

$$E_{L}\;\left(\mathrm{wt}\%\right)=\frac{w_{p}}{w_{t}}\times 100\%$$

where *w*_p_ and *w*_t_ are the mas of QC in micelles and total QC used in the preparation of micelles, respectively.

Release of QC loaded in polymer micelles was investigated as follows: 4 mL of QC loaded micelle solution was placed into a dialysis membrane (Spectra/Por dialysis membrane, MWCO 1000) and immersed into 70 mL of buffer solution (pH 1 and pH 9). The solutions were incubated at 37 °C. The samples were withdrawn periodically (1 mL) and replaced by 1 mL of fresh buffer solution. The concentration of released QC was investigated on the basis of UV-vis spectra.

## 3. Results and Discussion

### 3.1. Synthesis and Characterization of Rifampicin-Based ATRP Macroinitiator

**Figure 1:**
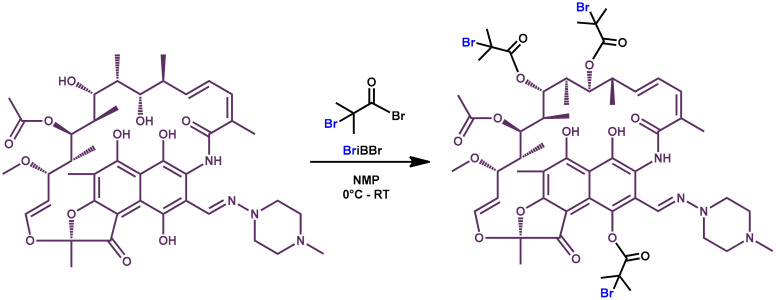
Synthetic route for the preparation of rifampicin-based ATRP macroinitiator (*M*_n_ = 1269.94, *Ð* = 1.19).

The synthetic route for the modification of rifampicin by introduction of polymer chains in its structure included two steps. Initially, a supramolecular ATRP macroinitiator was synthesized, followed by polymerization of acrylates from the rifampicin-based initiator, forming functional polymer chains. A rifampicin-based macromolecular initiator with bromine atoms (Rif-Br_3_, *M*_n_ = 1269.94, *Ð* = 1.19) was prepared by an esterification reaction of rifampicin with BriBBr, as shown in Figure 1.

The chemical structure of Rif-Br_3_ was confirmed using ^1^H NMR (Supplementary Figure S1b) analysis by assignment of the signals on the basis of ^1^H NMR spectra of rifampicin before modification (Supplementary Figure S1a) [62]. ^1^H NMR assignment of Rif-Br_3_: δ (ppm) = 0.23–0.07 (3H, CH_3_–, 1), 0.79–0.86 (3H, CH_3_–, 2), 0.95–1.14 (6H, CH_3_–, 3 + 4), 1.30–1.35 (1H, –CH–, 5), 1.65–1.70 (2H, –CH–, 6 + 7), 1.81–2.03 (27H, CH_3_–, 8 + 9 + 10 + –C(CH_3_)_2_), 2.19–2.40 (7H, CH_3_–, >N(CH_3_)–, –CH–, 11 + 12 + 13), 2.41–2.62 (4H, –CH_2_–, 14), 2.92–3.10 (7H, CH_3_–, –CH_2_–, 16 + 17), 3.10–3.22 (1H, –CH–, 15), 3.35–3.45 (1H, –CH–, 19), 4.08–4.26 (1H, –CH–, 21), 4.96–5.03 (1H, =CH–, 22), 5.05–5.27 (1H, –CH–, 23), 5.90–6.03 (1H, =CH–, 24), 6.17–6.23 (1H, =CH–, 25), 6.48–6.64 (1H, =CH–, 26), 6.65–6.78 (1H, =CH–, 27), 7.94–8.21 (1H, –N=CH–, 28), 12.90–13.86 (3H, –NH–, –OH, 30 + 31 + 32). After incorporation of bromine structure into rifampicin, new chemical shifts appear at δ = 1.81–2.03 (27H, 8 + 9 + 10 + –C(CH_3_)_2_), arising from the methyl protons adjacent to the bromine atom. The number of the brominated initiation sites in rifampicin-based ATRP macroinitiator was determined by the ratio between the integration areas of the proton localized in the imine group of rifampicin at the regions of δ = 7.94–8.21 ppm (1H) and the protons characteristic for methyl groups of incorporated bromine. Additionally, after modification of rifampicin by esterification reaction, ^1^H NMR spectra signals from three hydroxyl groups protons disapperared, which were at the regions of δ = 3.43–3.46 ppm, 3.58–3.64 ppm and 11.99–12.04 ppm in ^1^H NMR spectra of the substrate [63] (Supplementary Figure S1a). Chemical structure of Rif-Br_3_ was also confirmed by ^13^C NMR analysis as follows: δ (ppm) = 10.93 (C_1_), 13.57 (C_2_), 14.01 (C_3_), 19.62 (C_4_), 22.14 (C_5_), 22.94 (C_6_), 23.39 (C_7_), 23.91 (C_8_),27.80 (C_9_), 28.89 (C_10_), 29.15 (C_11_), 33.12 (C_12_), 38.70 (C_13_), 58.71 (C_14_), 67.33 (C_15_), 67.42 (C_16_), 67.66 (C_17_), 68.15 (C_18_), 68.60 (C_19_), 98.36 (C_20_), 100.81 (C_21_), 100.85 (C_22_), 101.61 (C_23_), 101.70 (C_24_), 101.71 (C_25_), 103.76 (C_26_), 103.79 (C_27_), 106.33 (C_28_), 107.28 (C_29_), 107.31 (C_30_), 107.37 (C_31_), 107.58 (C_32_), 107.81 (C_33_), 107.91 (C_34_), 128.78 (C_35_), 130.90 (C_36_), 132.39 (C_37_), 161.06 (C_38_), 161.17 (C_39_), 161.39 (C_40_), 167.70 (C_41_), 167.80 (C_41_), 171.04 (C_43_), 177.99 (C_44_) (Supplementary Figure S1c) [62]. The characteristic signals of the carbons in incorporated bromine structure were assigned (C_11_, C_20_ and C_43_). NMR spectra show residual impurities in the final product derived from solvents used in the synthesis and purification (H_2_O, NMP, acetone), however polar both parotic and aprotic solvents do not interfere with ATRP process [64,65] and such an inconsiderable amount does not affect the course of the reaction.

The structure of the brominated macromolecule was also analyzed by FT-IR analysis (Supplementary Figure S3). Significant differences in comparison to FT-IR spectra of unmodified rifampicin were observed. A new signal within 1700–1800 cm^−1^ and 1000–1150 cm^−1^ was identified, which are associated with C=O and C-O-C stretching vibrations in ester groups of incorporated bromine structure, respectively. Additionally, the bands at about 1220–1350 cm^−1^ related to C–H bending vibrations –CH_3_ groups of brominated molecules were noticed. There is a band in the 3100–3700 cm^−1^ region which is related to two unmodified –OH groups. The band is significantly less intense compared to the spectrum of the rifampicin substrate.

Additionally, UV-vis spectrophotometric measurements were recorded for unmodified and brominated rifampicin (Supplementary Figure S4). For rifampicin in THF, the UV-vis spectrum shows bands at 345 nm, 400 nm, and 465 nm. These bands decreased significantly in the spectrum after modification of a substrate with BriBBr molecules, proving successful incorporation of brominated initiation sites in rifampicin-based ATRP macroinitiator. Spectral changes and absorbance decrease are the results of the perturbation of the chromophore electrons of the rifampicin after incorporation of bromine molecules in its structure.

The initiation characteristics of rifampicin-inspired ATRP macroinitiator and the selection of an appropriate catalyst for polymerization was checked by an electrochemical investigation of different catalytic complexes (Cu^II^Br_2_/PMDETA, Cu^II^Br_2_/TPMA, Cu^II^Br_2_/TPMA*^2^) in the presence of the brominated structure. A series of CV measurements were carried out. As expected, CV of copper catalyst complexes demonstrated redox processes as a reduction of Cu^II^ to Cu^I^ and vice versa, evidenced by quasi-reversible peak couples with formal reduction potential *E*° ≈ (*E*_pc_ + *E*_pa_)/2 = −0.174 V vs. SCE, −0.237 V vs. SCE and −0.328 V vs. SCE, respectively, where *E*_pc_ is a cathodic peak potential and *E*_pa_ is an anodic peak potential (Supplementary Figure S5). More negative value of *E*° indicates a more active ATRP copper complex [58]. The addition of the brominated rifampicin to the reaction media resulted in the disappearance of the anodic peak and a significant decrease of the cathodic peak current. This phenomenon is described as the electrochemical catalytic process (EC’), where the catalyst electrochemically reduced to an active form (Cu^I^) rapidly reacts with alkyl halide macroinitiator (Rif-Br_3_). In consequence, Cu^I^ is oxidized back not electrochemically but by chemical reaction, and the reverse scan corresponding to oxidation of Cu^I^ to Cu^II^ does not occur [29,36,60]. The observation confirms the ability of the prepared rifampicin-based molecule to initiate the ATRP process.

For precise determination of the initiation functionality of the bromide structure, the rate constant of the EC’ (*k*_EC’_) with different catalyst complexes was determined. Activation reaction in ATRP process consists of a reversible electron transfer within the redox species followed by an irreversible reaction regenerating the starting form of catalyst, thus the foot-of-the-wave (FOWA) analysis developed by Savéant and co-workers [29,60,66,67] was used for determination of *k*_EC’_. TEMPO as a radical scavenge was used to make the overall process irreversible. The principle of this method is the normalization of the catalytic current (*i*) with respect to the peak current of the reversible one-electron reduction of the transition metal catalyst in the absence of the brominated structure (Rif-Br_3_) at the same scan rate, *i*_p_^0^ [66,67]. It results in the Equation:

(1) $$\frac{i}{i_{p}^{0}}=2.24\sqrt{\frac{k_{EC’}C_{\mathrm{Rif}\;-{\;\mathrm{Br}}_{3}}^{0}RT}{Fv}}\exp\left[-\frac{F}{RT}\left(E-E_{{\mathrm{Cu}}^{\mathrm{II}}/{\mathrm{Cu}}^{I}}^{0}\right)\right]$$

where $C_{\mathrm{Rif}\;-{\;\mathrm{Br}}_{3}}^{0}$ is initial concentration of Rif-Br_3_, *F* is the Faraday constant, *R* is the universal gas constant, *T* = 298 K, $E_{{\mathrm{Cu}}^{\mathrm{II}}/{\mathrm{Cu}}^{I}}^{0}$ is the half wave potential of the Cu^II^Br_2_/L catalyst complex, where the slope (*a)* of the plot (Equation (2)) $\frac{i}{i_{p}^{0}}\;\mathrm{vs}.\;\exp\left[-F\left(E-E_{{\mathrm{Cu}}^{\mathrm{II}}/{\mathrm{Cu}}^{I}}^{0}\right)/RT\right]$ give the value of *k*_EC_’.

(2) $$a=2.24\sqrt{\frac{k_{EC’}C_{\mathrm{Rif}\;-{\;\mathrm{Br}}_{3}}^{0}RT}{Fv}}$$

An average rate constant of EC’ is k_EC’_ = (2.73 ± 0.47) × 10^2^ M^−1^ s^−1^, k_EC’_ = (5.65 ± 0.97) × 10^3^ M^−1^ s^−1^, and k_EC’_ = (1.82 ± 0.20) × 10^4^ M^−1^ s^−1^, for Cu^II^Br_2_/PMDETA, Cu^II^Br_2_/TPMA and Cu^II^Br_2_/TPMA*^2^, respectively. The results indicate Cu^II^Br_2_/TPMA*^2^ as the most efficient catalyst in the reaction setup with Rif-Br_3_ as a supramolecular ATRP macroinitiator.

### 3.2. Synthesis of Rifmapicin-Based Macromolecules

Considering an electrochemical investigation of brominated rifampicin as an ATRP initiator with different catalytic complexes, Cu^II^Br_2_/TPMA*^2^, as the most active catalyst, was used for the synthesis of branched polymers, polymerizing *n*-butyl acrylate from rifampicin core (Table 1, entry 1, Supplementary Figure S7). Simplified electrochemically-mediated ATRP under constant potential conditions (applied potential, *E*_app_ = *E*_pc_ − 80 mV, Supplementary Figure S8a) was applied. As expected, initially a rapid decay of the cathodic current was observed, associated with the presence of Cu^II^ rapidly reduced to activator form (Cu^I^) at the working electrode surface upon the applied potential. Then, the current approaches constant value due to the adjustment of an equilibrium of the ratio between deactivator and activator form of the catalyst by the selected *E*_app_ (Supplementary Figure S8b) [36,68]. Initially, a linear first-order kinetics plot is observed, however, the polymerization slowed down considerably after 4.5 h of the process, reaching monomer conversion of 48% (Supplementary Figure S8c). Despite the stopping of the polymerization in the last hour, the last kinetics point of *M*_n_ vs. monomer conversion plot deviates from linearity towards higher molecular masses, indicating inter- and intramolecular coupling reaction, thus the formation of uncontrolled high molecular weight polymer structures (Supplementary Figure S8d). It results in a broad molecular weight distribution of the final product (*M*_w_/*M*_n_ = 1.63, Supplementary Figure S8e).

**Figure 2:**
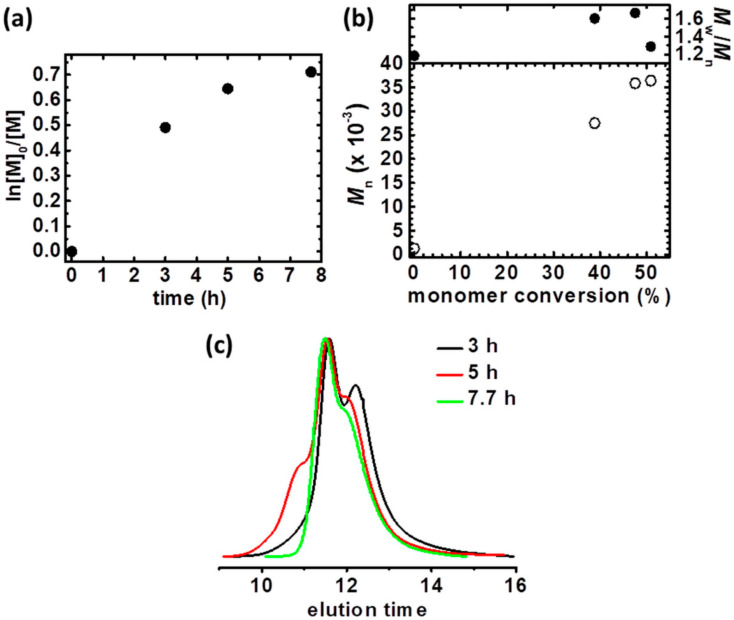
(**a**) First-order kinetic plot of monomer conversion vs. polymerization time for constant potential electrolysis in the preparation of Rif-(P*n*BA-Br)_3_ macromolecules, (**b**) *M*_n_ and *M*_w_/*M*_n_ vs. monomer conversion and (**c**) GPC traces of *n*BA polymerization and their evolution over reaction time. Table 1, entry 2.

Polymerization with the use of unsubstituted TPMA as a ligand was conducted (Table 1, entry 2, Supplementary Figure S9a,b). Similarly, as with Cu^II^Br_2_/TPMA*^2^ catalyst complex, the first-order kinetics plot deviates from linearity, indicating for gradual stopping of the polymerization process, receiving comparable monomer conversion (conv = 51%, Figure 2a). However, the received rifampicin-based macromolecules were characterized by lower dispersity (*M*_w_/*M*_n_ = 1.29), and the *M*_n_ vs. monomer conversion plot does not indicate the coupling reaction by inter- and intramolecular reaction (Figure 2b). The molecular weight of the two last kinetics samples insignificantly changes, proving the formation of the new homopolymer chains in the reaction setup, suggesting transfer to solvent, monomer, or more attributed to preparative electrolysis – to an electrolyte. A low molecular weight shoulder is observed in the GPC trace of polymerization product (Figure 2c). Despite the lower activity comparing to Cu^II^Br_2_/TPMA*^2^, catalyst complex with TPMA provided more controlled polymerization of rifampicin-based macromolecules. The additional electron-donating groups in TPMA*^2^ structures increase the reduction properties of the corresponding activator complexes (Cu^I^), shifted the equilibrium toward stabilization of the oxidized catalyst form, as proved by calculation of Cu^I^/Cu^II^ ratio (0.02 vs. 31.06, compare [Cu^I^]/[Cu^II^], Supplementary Table S1, entries 1 and 2) [58]. It results in faster polymerization (*k*_p_^app^ = 0.133 vs. 0.109, compare *k*_p_^app^, Table 1, entries 1 and 2). Although the use of Cu^II^Br_2_/TPMA*^2^ provided a synthesis of more controlled linear structure comparing to Cu^II^Br_2_/TPMA ligand [58], substituted with electron-donating groups pyridine ligand is too active for the preparation of architecture macromolecules presented in this paper, losing control during synthesis, resulting in coupling reactions, and thus in high dispersity and a higher theoretical dead chain fraction value (DCF_theo_ = 0.26 vs. 0.12, compare DCF_theo_, Supplementary Table S3, entries 1 and 2).

**Figure 3:**
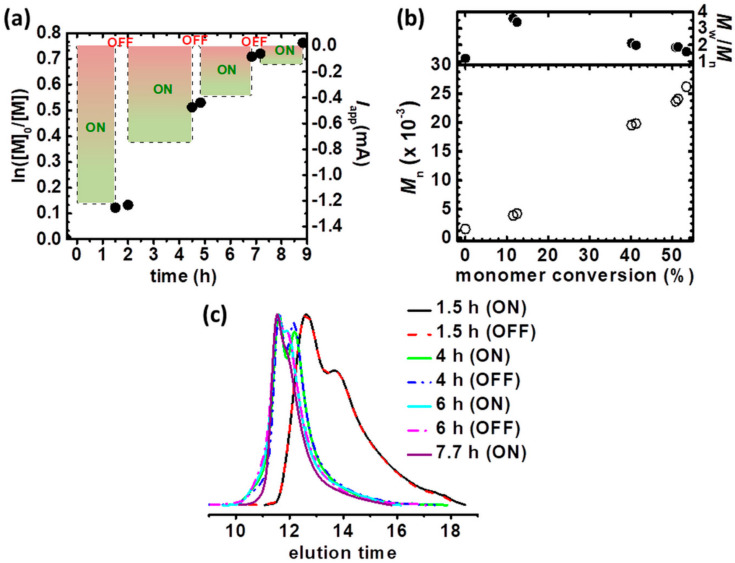
(**a**) First-order kinetic plot of monomer conversion vs. polymerization time for temporally control constant current electrolysis in the preparation of Rif-(P*n*BA-Br)_3_ macromolecules, (**b**) *M*_n_ and *M*_w_/*M*_n_ vs. monomer conversion and (**c**) GPC traces of *n*BA polymerization and their evolution over reaction time. Table 1, entry 3.

To simplify an electrochemical cell, the current constant conditions were applied to conduct the polymerization of rifampicin-based polymers (Table 1, entry 3). Electrolysis under galvanostatic conditions is privileged from both, laboratory and potential scaling the reaction setup to industrial scale. An electrochemical cell is simplified by the elimination of RE, and a potentiostat/galvanostat can be replaced by a simple direct-current power supply [27,36,52,55]. The current profile was established on the basis of potentiostatic electrolysis as *I* = *Q*/t (*Q*–electric charge, t–polymerization time) (Table 1, entry 2), and consisted of 4 current stages (applied current, *I*_app_ = 1.22 mA, 0.75 mA, 0.39 mA and 0.14 mA, respectively) to appropriately adjust the chronoamperometric curve (Supplementary Figure S10a). Additionally, temporal control over the polymerization was implemented, stopping and restarting the polymerization by switching between ON (*I*_app_) and OFF (*I*_app_ = 0 A) stages, respectively. This procedure verifies the livingness of the electrochemical-mediated process and provides control over the molecular weight of polymer chains. The observed trend of the first-order kinetics plot (Figure 3a), *M*_n_ vs. monomer conversion (Figure 3b) and GPC traces of the samples withdrawn periodically during synthesis, clearly proved the living characteristics of preparative electrolysis, namely while the current value dropped to 0 mA, the polymerization of the monomer and thus an increase of polymer molecular weight was stopped. The temporally control galvanostatic electrolysis provided the final product with broader molecular weight distribution (*M*_w_/*M*_n_ = 1.29 vs. 1.59, compare *M*_w_/*M*_n_, Table 1, entries 2 and 3, respectively). Despite the DCF_theo_ value below 1%, after restarting the polymerization, the chain-and functionality of growing polymers was slightly lost, resulting in the product with higher dispersity comparing to the macromolecules received in the electrolysis under potentiostatic conditions without temporal control stages.

**Figure 4:**
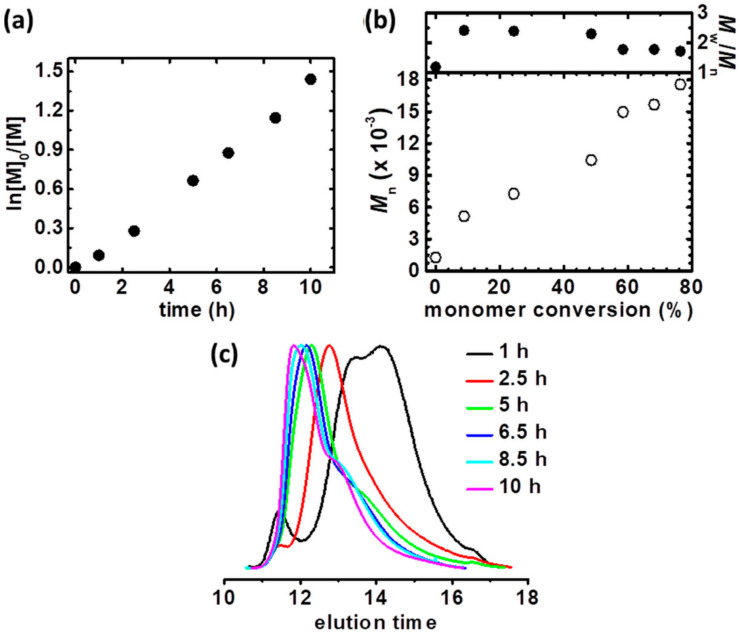
(**a**) First-order kinetic plot of monomer conversion vs. polymerization time for constant potential electrolysis in the preparation of Rif-(P*t*BA-Br)_3_ macromolecules, (**b**) *M*_n_ and *M*_w_/*M*_n_ vs. monomer conversion and (**c**) GPC traces of *t*BA polymerization and their evolution over reaction time. Table 1, entry 4.

Motivated by better control during the polymerization catalyzed by Cu^II^Br_2_/TPMA, *t*BA as a precursor of polyelectrolytes, was polymerized from rifampicin-based ATRP macroinitiator via *se*ATRP under potentiostatic conditions (Table 1, entry 4, Supplementary Figure S11a,b). Linear characteristics of both first-order kinetics plot (Figure 4a) and an increase of polymer molecular weight as a function of monomer conversion (Figure 4b,c) was observed. However, the final product is characterized by broader molecular weight distribution comparing to Rif-(P*n*BA-Br)_3_ (*M*_w_/*M*_n_ = 1.71 vs. 1.29, compare *M*_w_/*M*_n_, Table 1, entries 2 and 4, respectively). It is directly connected with the higher macroinitiator concentration in the polymerization of *t*BA. According to the equation describing the dispersity *M*_w_/*M*_n_ = 1 + (*k*_p_[P-X]/*k*_da_[Cu^II^])(2/p − 1) (where *k*_p_ is rate constant of propagation, [P-X]–macroinitiator concentration, *k*_da_–rate constant of deactivation, [Cu^II^]–copper (II) concentration and p–monomer conversion) [69,70], higher [P-X] provides macromolecules with higher dispersity.

**Figure 5:**
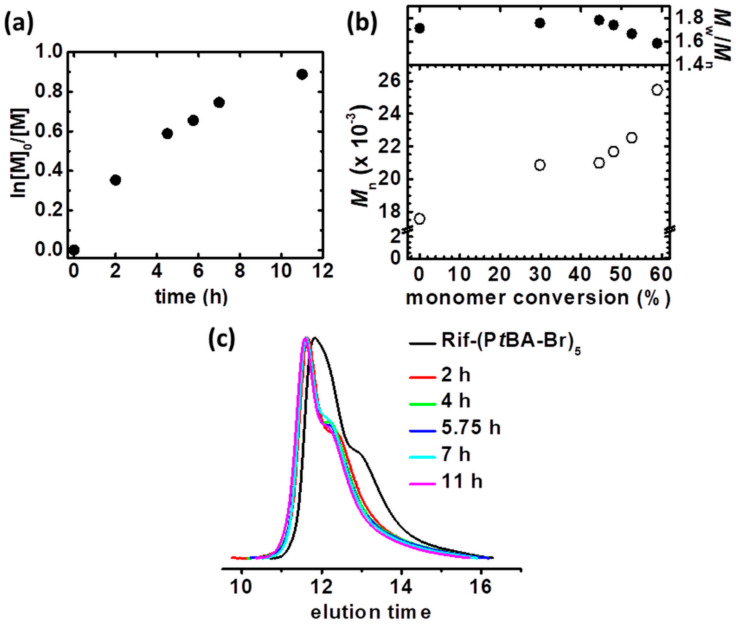
(**a**) First-order kinetic plot of monomer conversion vs. polymerization time for constant potential electrolysis in the preparation of Rif-(P*t*BA-*b*-P*t*BA-Br)_3_ macromolecules, (**b**) *M*_n_ and *M*_w_/*M*_n_ vs. monomer conversion and (**c**) GPC traces of *t*BA polymerization and their evolution over reaction time. Table 1, entry 5.

Chain extension experiment was conducted to examine chain-end functionality of Rif-(P*t*BA-Br)_3_ (Table 1, entry 5, Supplementary Figure S11a,b). Additional P*t*BA polymer block was incorporated into the side chains of rifampicin-based macromolecules applied as an ATRP macromolecular initiator (*M*_n,theo_ = 30100, *M*_w_/*M*_n_ = 1.71). The polymerization process was characterized by a linear semi-logarithmic kinetics plot (Figure 5a) and an increase in polymers MW during polymerization (Figure 5b,c) was observed. It results in a final polymer product with lower dispersity compared to the first P*t*BA block *M*_w_/*M*_n_ = 1.58 vs. 1.71, compare *M*_w_/*M*_n_, Table 1, entries 4 and 5, respectively). The experiment proved the preservation of chain-end fidelity of the Rif-(P*t*BA-Br)_3_ macroinitiator and affirmed the validity of theoretical DCF value calculations (DCF_theo_ < 1%).

The characteristic element of *se*ATRP equipment is aluminum as a counter electrode, which simplifies the electrochemical cell by direct immersion of the electrode into the reaction mixture, it is simple to prepare and scale, cost-effective and energy-efficient (minimizes the ohmic drop) process [54]. Despite the negative standard potential value (*E*_0_ = 1.66 V vs. SHE in water) of aluminum [71] and the potential for reduction of Cu^II^ to Cu^I^, it does not participate in the redox processes of the catalyst due to a passivation process (covering by stable oxidized layers). As CE is directly immersed in a reaction medium, both reaction mixture and final polymer contain an insignificant amount of aluminum (Supplementary Table S2). The content of aluminum in the reaction mixture is 48.9–128.0 ppm, increasing with the higher value of *Q*, while the final polymer samples are contaminated with 11.7–48.9 ppm of the aluminum, due to the purification procedures (through a neutral alumina column and precipitation into methanol/water solution).

The structure of formed acrylates-based polymers was confirmed by ^1^H NMR analysis (Supplementary Figures S13–S15). ^1^H NMR spectrum, shown in Figure S13 proved the polymerization of *n*BA from the rifampicin-based macromolecule by preparative electrolysis under potentiostatic conditions (Table 1, entry 3). The chemical shifts characteristic for P*n*BA chains were assigned: δ (ppm) = 0.88–1.00 (3H, CH_3_–, d), 1.30–1.98 (6H, –CH_2_–CH_2_–, –CH_2_–, b, d and β, respectively), 2.19–2.47 (1H, –CH–, α) and 3.85–4.21 (2H, –OCH_2_–, a) [72]. Meanwhile, Supplementary Figure S14 and S15 show the ^1^H NMR spectrum for rifampicin-based polymers with P*t*BA side chains (Table 1, entry 4). The chemical shifts for *t*BA units were assigned: δ (ppm) = 1.35–1.95 (11H, CH_3_–, –CH_2_–, a + β, respectively) and 2.10–2.38 (1H, –CH–, α) [73]. The identified chemical shifts attributed to the characteristic groups of the *n*BA and *t*BA units indicate the presence of polymer chains.

Rifampicin-based macromolecules with P*t*BA side chains were subjected to acid solvolysis to detached the homopolymer chains (Supplementary Table S4, Figure S16), and therefore the apparent molecular weights of cleaved chains and initiation efficiency (ƒ_i_) were determined. P*t*BA arms were characterized by low molecular weight and narrow molecular weight distribution (*M*_w_/*M*_n_ = 1.20 for P*t*BA, and *M*_w_/*M*_n_ = 1.42 for P*t*BA-*b*-P*t*BA polymer chains), however the low initiation efficiency was received (ƒ_i_ = 42–53%), suggesting disruption of the deactivation process followed by the loss of control as the reaction proceeded.

### 3.3. Preparation of pH-Responsive Rifampicin-Based “Smart” Materials

Stimuli-responsive polymer materials with rifampicin core and poly(acrylic acid) side chains were received by selective hydrolysis of the P*t*BA block (final samples synthesized according to Table 1, entries 4 and 5). The successfully received PAA structures were confirmed by ^1^H NMR analysis, comparing the spectra before and after hydrolysis (Supplementary Figures S14 and S15–P*t*BA side chains, and Figures S17 and S18–PAA side chains, respectively). After modification, the disappearance of the characteristic strong peak for methyl groups protons of *t*BA units at ~1.44 ppm is visible. Therefore, the chemical shift at ~12.00 ppm attributed to hydroxyl groups of prepared AA moieties occurs [74], proving the successfully conducted hydrolysis of P*t*BA polymer chains.

FT-IR analysis additionally confirmed the presence of acidic groups in the samples after hydrolysis. In Supplementary Figures S19a and S20a, the characteristic bands for P*t*BA are clearly visible. Stretching C–H vibrations from the ubiquitous –CH_3_ groups in polyacrylates give a significant band in the 2950–3050 cm^−1^ region. Stretching C=O and C–O–C vibrations in ester groups give the bands in 1700–1800 cm^−1^ and 1050–1200 cm^−1^, respectively. Typical absorption bands for P*t*BA is also located within 1380–1450 cm^−1^ region, corresponding to C–H bending vibrations from –CH_3_ groups of *t*BA. After the transformation of *t*BA moieties into AA groups, significant differences in the 3000–3700 cm^−1^ region is visible (Supplementary Figures S19b and S20b). A strong absorption between 2500 and 3600 cm^−1^ of carboxyl groups appeared. Additionally, the bands located in 1050–1200 cm^−1^ characteristic for stretching C–O–C vibrations in ester groups diapered.

The hydrophilicity, and thus an additional confirmation of *t*BA to AA unit hydrolysis, was provided by contact angles for polar (water) and non-polar (diiodomethane) liquid measurements. The results were subsequently applied for calculation of free surface energy values according to the Owens–Wendt method, considering the disruption of intermolecular bonds of a prepared surface (Supplementary Figure S21, Table S5) [31,75,76]. Considering initial studies covering contact angles examination, the decrease of θ for water as a polar liquid is observed, indicating PAA-based coatings as more hydrophilic. Hydrolysis of hydrophobic P*t*BA segments results in a significant increase in both dispersive and polar part values, and consequently, the total FSE parameter was higher for PAA-based coating materials. This phenomenon is strongly connected with ubiquitous hydroxyl groups of AA units. The results clearly indicate the hydrophilic properties of smart materials composed of AA moieties.

**Figure 6:**
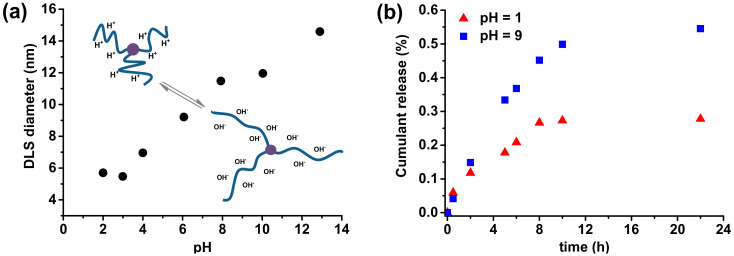
(**a**) DLS diameters of rifampicin-based macromolecules with PAA side chains (Table 1, entry 5 after acidic hydrolysis) at varying pH with a schematic representation of possible polymers configurations, (**b**) Quercetin release profile from rifampicin-based micelles in different pH buffer solutions at 37 °C.

Poly(acrylic acid) is a weak polyelectrolyte with a substantial portion of the negatively charged units at basic conditions when the carboxylic group loses a proton. Dynamic light scattering was performed to analyze the pH-responsiveness of the rifampicin-based macromolecules with PAA side chains, by determination of the hydrodynamic diameter at different degrees of ionization, adjusted by variation in pH value (Supplementary Figure S22). The response of PAA side chains for pH changes is shown in Figure 6a (Supplementary Table S6). An initial rifampicin-based polymer solution was prepared at strongly alkaline conditions (pH = 12.9), and then it was gradually decreased to pH = 2.

At highly alkaline pH values, when the solution contains high hydroxyl ions concentration, the acid segments of AA units are completely negatively charged. It results in the stretched side chains of rifampicin-based polymers, which shows a maximum in hydrodynamic diameter of 14.59 nm. With the decrease in pH value, the degree of ionization also decreased, causing the coiled conformation of PAA side chains, and hence a smaller DLS diameter is observed. Considering pH responsiveness of prepared rifampicin-based macromolecules with PAA side chains, they could be used as a carrier of active substances to achieve control release in different pH values. Quercetin, a flavonoid with many therapeutic activities, was encapsulated into the rifampicin-based micelles (loading efficiency, *E*_L_ = 60%) in aqueous solution by a dialysis method, followed by a pH-dependent release process of QC from micelles was investigated by UV-vis spectrometry (Figure 6b). At highly acidic conditions (pH = 1), when PAA segments are coiled, and thus encapsulate the substances, the micelles showed relatively low release level, only reaching 28% of released QC after 22 hours. Meanwhile, in the buffer solution at pH 9, the release rate of QC rapidly increases and finally reached 55% of the QC released from the micelles.

## 4. Conclusions

Rifampicin was successfully modified to receive stimuli-responsive polymer materials, using a two-step synthetic route according to “grafting from” strategy. Initially, the supramolecular initiator was prepared by an esterification reaction of rifampicin and BriBBr, receiving ATRP macroinitiator with three bromide initiation sites, proved by ^1^H NMR analysis, FT-IR spectrum. Electrochemical characterization of rifampicin-based macroinitiator provided by a series of CV measurements proved the initiation characteristics of the prepared molecule and enabled the selection of an appropriate catalytic complex for polymerization, determining rate constants of the electrochemical catalytic process (*k*_EC’_) for each catalyst in the presence of the rifampicin-based initiator (*k*_EC’_ = (2.73 ± 0.47) × 10^2^ M^−1^ s^−1^, *k*_EC’_ = (5.65 ± 0.97) × 10^3^ M^−1^ s^−1^, and *k*_EC’_ = (1.82 ± 0.20) × 10^4^ M^−1^ s^−1^, for Cu^II^Br_2_/PMDETA, Cu^II^Br_2_/TPMA and Cu^II^Br_2_/TPMA*^2^, respectively). Preparative electrolysis under constant potential conditions and temporally controlled galvanostatic conditions was performed to receive polymer materials with rifampicin core and acrylates side chains (P*n*BA and P*t*BA). Despite the lower activity of Cu^II^Br_2_/TPMA in comparison to Cu^II^Br_2_/TPMA*^2^, catalyst complex with unsubstituted pyridine provided a more controlled polymerization of rifampicin-based macromolecules (*M*_w_/*M*_n_ = 1.29). Substituted with electron-donating groups pyridine ligand is too active for the preparation of architecture macromolecules presented in this paper, losing control during synthesis, resulting in coupling reactions, and thus in high dispersity. Stimuli-responsive polymer materials sensitive to pH changes were prepared by the transformation of *t*BA into AA moieties by acidic hydrolysis, proved by ^1^H NMR, FT-IR and contact angles, followed by free surface energy analysis of prepared coatings. The pH-mediated behavior of PAA-based macromolecules was investigated by dynamic light scattering (DLS), determining a hydrodynamic radius of polymers upon pH changes. The PAA side chains were stretched (maximum in hydrodynamic diameter) at highly alkaline pH values, thus potentially released a substance, and the coiled conformation of PAA side chains (significant decrease in hydrodynamic diameter) in the acidic conditions was observed, potentially encapsulation of the drug. The loading followed by the controlled release of quercetin as a model active substance in the rifampicin-based micelles proved the pH-dependent behavior of the prepared polymer material. Encapsulation of QC in a buffer solution with highly acidic conditions and release in basic solution was observed.

## Figures and Tables

**Table 1:** Polymerization of acrylates from rifampicin-based ATRP macroinitiator via *se*ATRP ^a^.

Entry	[Monomer]_0_/[Initiator]_0_/[CuL]	Ligand	Monomer	Initiator	[CuL]	Conv ^b^ (%)	*k*_p_^app b^ (h^−1^)	DP_n_,_theo_^b^ (per arm)	*M*_n,theo_^c^ (×10^−3^)	*M*_n,app_^d^ (×10^−3^)	*M*_w_/*M*_n_^d^	*d*_volume_^e^ (nm)
1	270/1/0.11	TPMA*^2^	*n*BA	Rif-Br_3_	400	48	0.133	130	51.1	26.1	1.63	12 ± 1
2	270/1/0.11	TPMA	*n*BA	Rif-Br_3_	400	51	0.109	137	54.1	36.4	1.29	16 ± 1
3 ^f^	270/1/0.11	TPMA	*n*BA	Rif-Br_3_	400	53	0.095 ^g^	144	56.1	26.2	1.59	15 ± 2
4	101/1/0.04	TPMA	*t*BA	Rif-Br_3_	400	76	0.137	77	30.1	17.6	1.71	9 ± 1
5	182/1/0.07	TPMA	*t*BA	Rif-(P*t*BA-Br)_3_ from entry 4	400	59	0.097	107	72.1	25.6	1.58	14 ± 1

General reaction conditions: *T* = 50 °C; *V*_tot_ = 16 mL (except entry 1: *V*_tot_ = 25 mL and entry 5: *V*_tot_ = 14 mL); entry 1: [*n*BA]_0_ = 1.10 M, [Rif-Br_3_]_0_ = 1.35 mM calculated per 3 Br initiation sites, [Cu^II^Br_2_/TPMA*^2^]_0_ = 0.44 mM; entry 2 and 3: [*n*BA]_0_ = 2.19 M, [Rif-Br_3_]_0_ = 2.71 mM calculated per 3 Br initiation sites, [Cu^II^Br_2_/TPMA]_0_ = 0.88 mM; entry 4: [*t*BA]_0_ = 2.05 M, [Rif-Br_3_]_0_ = 6.74 mM calculated per 3 Br initiation sites, [Cu^II^Br_2_/TPMA]_0_ = 0.82 mM; entry 5: [*t*BA]_0_ = 1.07 M, [Rif-(P*t*BA-Br)_3_]_0_ = 1.95 mM calculated per 3 Br initiation sites, [Cu^II^Br_2_/TPMA]_0_ = 0.43 mM; Constant potential *se*ATRP (WE = Pt, CE = Al, RE = SCE): entry 1, 2, 4 and 5; *se*ATRP under temporally-controlled galvanostatic conditions (WE and CE without RE): entry 3; ^a^ *E*_app_ were selected based on CV analysis of Cu^II^Br_2_/L catalytic complexes (Figures S8a, S9a, S11a and S12a in SI, respectively); ^b^ monomer conversion, apparent rate constant of propagation (*k*_p_^app^) and apparent theoretical degree of polymerization of monomer unit per arm (DP_n,theo_) were determined by NMR, *k*_p_^app^ calculated as a slope of the curve ln[M]_0_/[M] = *f(*t) illustrated in the Figure 2a, Figure 3a, Figure 4a and Figure 5a, and Supplementary Figure S8c [27,30]; ^c^ *M*_n,th_ = ([monomer]_0_/[initiator]_0_) × conversion × *M*_monomer_ + *M*_initiator_; ^d^ apparent *M*_n_ and *M*_w_/*M*_n_ were determined by GPC; ^e^ volume mean diameter (*d*_volume_) of polymer products measured by DLS after purification (Supplementary Figures S8f, S9c, S10b, S11c, S12c); ^f^ *I*_app_ = 1.22 mA, 0.75 mA, 0.39 mA and 0.14 mA for each step; ^g^ only for the “ON” stages.

## Supplementary Materials

The following are available online at https://www.mdpi.com/1996-1944/13/17/3843/s1, Figure S1: ^1^H NMR of (a) rifampicin (CDCl_3_); (b) ^1^H NMR and (c) ^13^C NMR of Rif-Br_3_ supramolecular initiator (*M*_n_ = 1269.94, *Ð* = 1.19) after purification (CDCl_3_), Figure S2: GPC trace of Rif-Br_3_ macroinitiator; Figure S3: FT-IR characterization of (a) rifampicin and (b) Rif-Br_3_ macroinitiator, Figure S4: UV-vis spectrum of rifampicin and Rif-Br_3_ macroinitiator in THF, Figure S5: Cyclic voltammogram of 0.8 mM Cu^II^Br_2_/L in DMF containing 0.2 M TBAP in the absence (black line) and in the presence of 9.8 mM Rif-Br_3_ (red line) recorded at *v* = 0.1 V⋅s^−1^, where L (ligand) is (a) PMDETA, (b) TPMA and (c) TPMA*^2^ Figure S6: Cyclic voltammograms of 0.8 mM Cu^II^Br_2_/L in DMF recorded at a different scan rates (given next to the curves) in the presence of 9.8 mM Rif-Br_3_ (3 Br molecules) and 39.9 mM TEMPO; the current was normalized with respect to the peak current (*i*_p_^0^) recorded in the absence of Rif-Br_3_, where L (ligand) is (a) PMDETA, (**c**) TPMA and (**e**) TPMA*^2^; Foot-of-the-wave analysis of the catalytic peak to determine *k*_a_, the slope a of the plots of *i*/*i*_p_^0^ vs. exp[-*F*(*E* − *E*_Cu(II)/Cu(I)_^0^)/*RT*]: $a=2.24\sqrt{\frac{k_{a}C_{A}^{0}RT}{Fv}}$, where *i*—catalytic current, *i*_p_^0^—reversible one-electron reduction of the copper catalyst complex in the absence of Rif-Br_3_, *C*_A_^0^—initial Rif-(P*n*BA-Br)_3_ concentration, *F*—Faraday constant, *R*—gas constant, *T* = 298 K, $E_{{\mathrm{Cu}}^{\mathrm{II}}/{\mathrm{Cu}}^{I}}^{0}$ is the half wave potential of the Cu^II^Br_2_/L, using (b) PMDETA, (d) TPMA and (e) TPMA*^2^ as a ligand, Figure S7: Synthetic route for the preparation of rifampicin-based macromolecules with acrylates (P*n*BA and P*t*BA) and poly(acrylic acid) (PAA) side chains, Figure S8: (a) Cyclic voltammogram of 0.44 mM Cu^II^Br_2_/TPMA*^2^ in 15% (*v*/*v*) *n*BA/DMF ([*n*BA]_0_ = 1.10 M) containing 0.2 M TBAP in the absence (black line) and in the presence of 1.35 mM Rif-Br_3_ (red line) recorded at *v* = 0.1 V⋅s^−1^, (b) current profile vs. time for the polymerization of *n*BA from Rif-Br_3_, (c) First-order kinetic plot of monomer conversion vs. time, (d) *M*_n_ and *M*_w_/M_n_ vs. monomer conversion, (e) GPC traces of *n*BA polymerization and their evolution over reaction time, (f) DLS hydrodynamic size distributions by volumeof Rif-(P*n*BA-Br)_3_. Table 1, entry 1, Figure S9: (a) Cyclic voltammogram of 0.88 mM Cu^II^Br_2_/TPMA in 30% (*v*/*v*) *n*BA/DMF ([*n*BA]_0_ = 2.19 M) containing 0.2 M TBAP in the absence (black line) and in the presence of 2.71 mM Rif-Br_3_ (red line) recorded at *v* = 0.1 V⋅s^−1^, (b) current profile vs. time for the polymerization of *n*BA from Rif-Br_3_, (c) DLS hydrodynamic size distributions by volume of Rif-(P*n*BA-Br)_3_. Table 1, entry 2, Figure S10: (a) Current profile vs. time for the polymerization of *n*BA from Rif-Br_3_ under constant potential conditions and the determined current steps for constant current electrolysis (c) DLS hydrodynamic size distributions by volume of Rif-(P*n*BA-Br)_3_. Table 1, entry 3, Figure S11: (a) Cyclic voltammogram of 0.82 mM Cu^II^Br_2_/TPMA in 30% (*v*/*v*) *t*BA/DMF ([*t*BA]_0_ = 2.05 M) containing 0.2 M TBAP in the absence (black line) and in the presence of 6.74 mM Rif-Br_3_ (red line) recorded at *v* = 0.1 V⋅s^−1^, (b) current profile vs. time for the polymerization of *t*BA from Rif-Br_3_, (c) DLS hydrodynamic size distributions by volume of Rif-(P*t*BA-Br)_3_. Table 1, entry 4, Figure S12: (a) Cyclic voltammogram of 0.43 mM Cu^II^Br_2_/TPMA in 16% (*v*/*v*) *t*BA/DMF ([*t*BA]_0_ = 1.07 M) containing 0.2 M TBAP in the absence (black line) and in the presence of 1.95 mM Rif-(P*t*BA-Br)_3_ (red line) recorded at *v* = 0.1 V⋅s^−1^, (b) current profile vs. time for the polymerization of *t*BA from Rif-(P*t*BA-Br)_3_, (c) DLS hydrodynamic size distributions by volume of Rif-(P*t*BA-*b*-P*t*BA-Br)_3_. Table 1, entry 5, Figure S13: ^1^H NMR spectrum of Rif-(P*n*BA-Br)_3_ polymers (*M*_n_ = 56100, *Ð* = 1.59) after purification (in CDCl_3_). Table 1, entry 3, Figure S14: ^1^H NMR spectrum of Rif-(P*t*BA-Br)_3_ polymers (*M*_n_ = 30100, *Ð* = 1.71) after purification (in CDCl_3_). Table 1, entry 4, Figure S15: ^1^H NMR spectrum of Rif-(P*t*BA-*b*-P*t*BA-Br)_3_ polymers (*M*_n_ = 72100, *Ð* = 1.58) after purification (in CDCl_3_). Table 1, entry 5, Figure S16: GPC traces of (a) Rif-(P*t*BA-Br)_3_ (Table 1, entry 4) and the corresponding cleaved P*t*BA arms, and (b) Rif-(P*t*BA-*b*-P*t*BA-Br)_3_ (Table 1, entry 5) and the corresponding cleaved P*t*BA-*b*-P*t*BA arms, Figure S17: ^1^H NMR spectrum of Rif-(PAA-Br)_3_ polymers after purification (in DMSO-*d*_6_). Table 1, entry 4, Figure S18: ^1^H NMR spectrum of Rif-(PAA*-b-*PAA-Br)_3_ polymers after purification (in DMSO-*d*_6_). Table 1, entry 5; Figure S19: FT-IR characterization of (a) Rif-(P*t*BA-Br)_3_ (Table 1, entry 4) and (b) Rif-(PAA-Br)_3_, Figure S20: FT-IR characterization of (a) Rif-(P*t*BA-*b*-P*t*BA-Br)_3_ (Table 1, entry 5) and (b) Rif-(PAA *b*-PAA-Br)_3_, Figure S21: Water contact angle images of (a) Rif-(P*t*BA-Br)_3_ (Table 1, entry 4) and (b) corresponding Rif-(PAA-Br)_3_, and diiodomethane contact angle images of (c) Rif-(P*t*BA-Br)_3_ (Table 1, entry 4) and (d) corresponding Rif-(PAA-Br)_3_, Figure S22: DLS hydrodynamic size distributions by volume of Rif-(P*t*BA-*b*-P*t*BA-Br)_3_ in different pH, Table S1: Calculation of Cu^I^/Cu^II^ ratio for the preparation of rifampicin-based macromolecules, Table S2: Theoretical Al^3+^ concentration in solution and polymer by monomer conversion, Table S3: Calculation of theoretical Dead Chain Fraction (DCF_theo_) for polymerization of acrylates at low copper catalyst loading, Table S4: Results of the detaching of polymer arms from rifampicin-based macromolecules, Table S5: Experimental values of contact angles, parameters of free surface energy (FSE) as calculated by Owens-Wendt method for rifampicin-based polymer coatings, Table S6: Volume mean diameter of rifampicin-based macromolecules at varying pH.
