# Supplementary material for: Stimuli-Responsive Rifampicin-Based Macromolecules
Source: Materials (Basel). 2020 Aug 31;13(17):3843. doi: 10.3390/ma13173843 (PMC7503961; doi:10.3390/ma13173843)
Supplement: Supplementary file 1 [file materials-13-03843-s001.pdf]

materials

Supplementary Materials

Stimuli-Responsive Rifampicin-Based Macromolecules

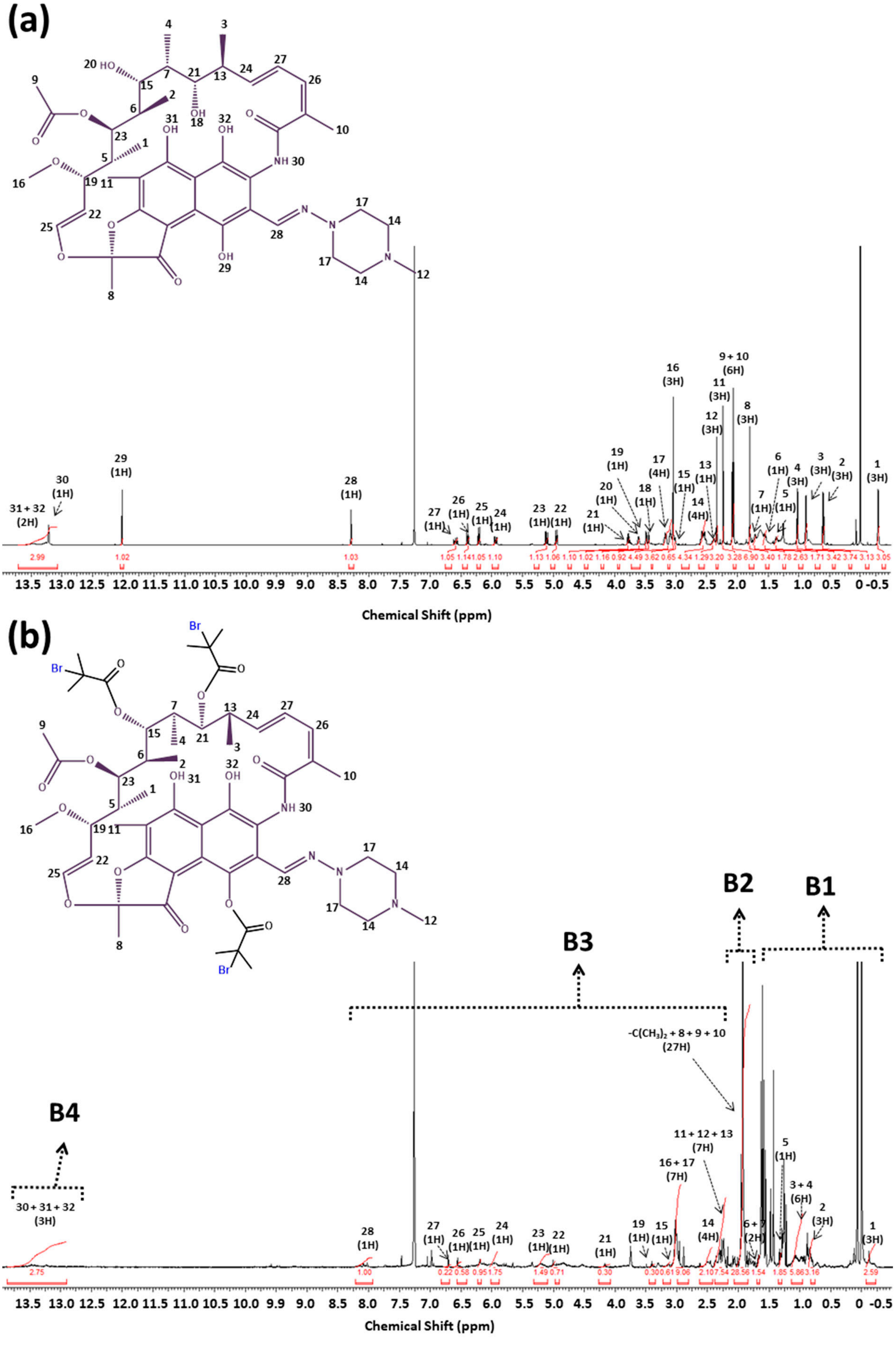
(a)
Chemical Shift (ppm)
(b)
B1
B2
B3
B4
-C(CH3)2 + 8 + 9 + 10
(27H)
11 + 12 + 13
(7H)
16 + 17
(7H)
30 + 31 + 32
(3H)
31 + 32
(2H)
9 + 10
(6H)
Chemical Shift (ppm)

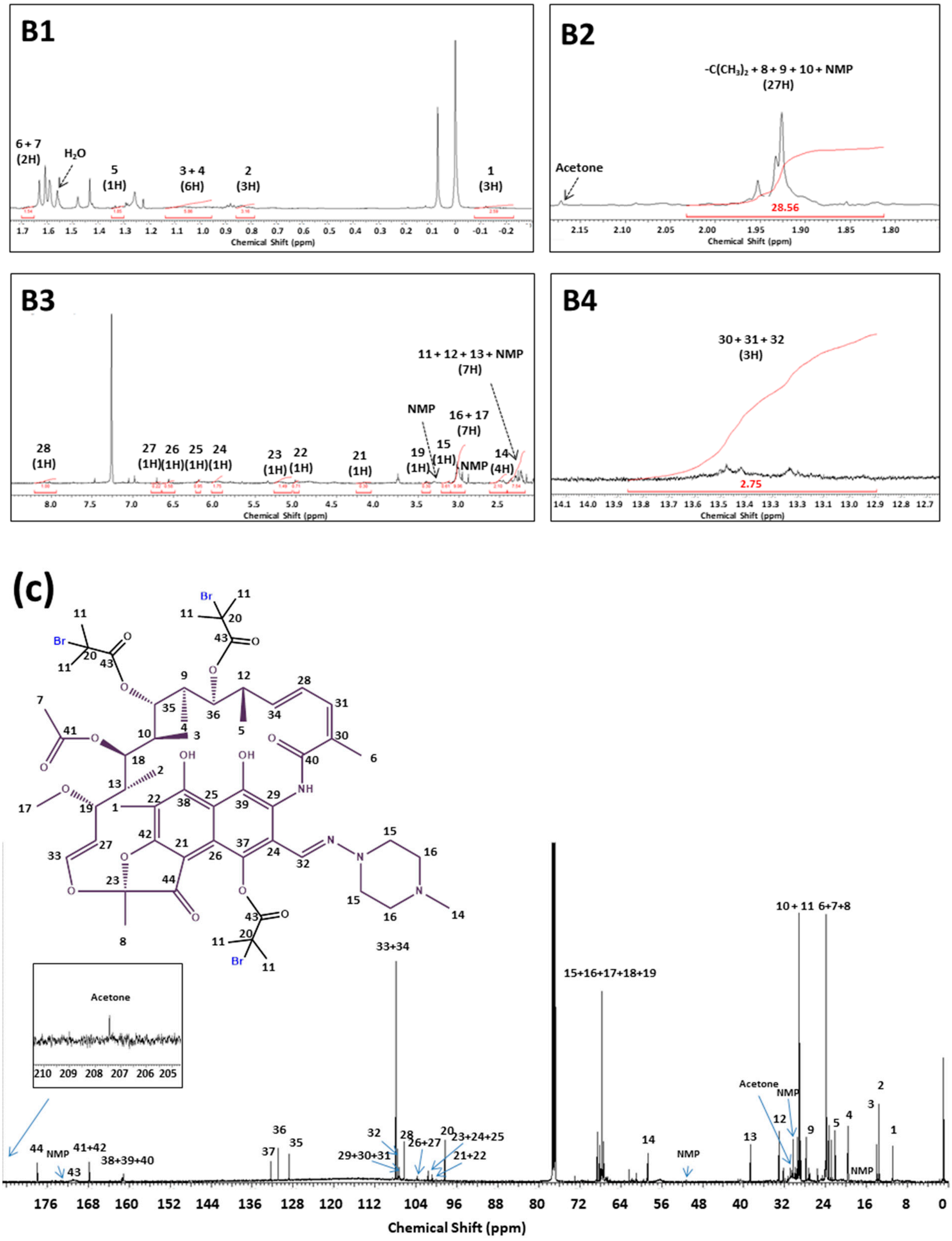

Figure S1. ^{1}H NMR of (**a**) rifampicin (CDCl$_3$); (**b**) ^{1}H NMR and (**c**) ^{13}C NMR of Rif-Br$_3$ supramolecular initiator (M_n = 1269.94, $Đ$ = 1.19) after purification (CDCl$_3$).

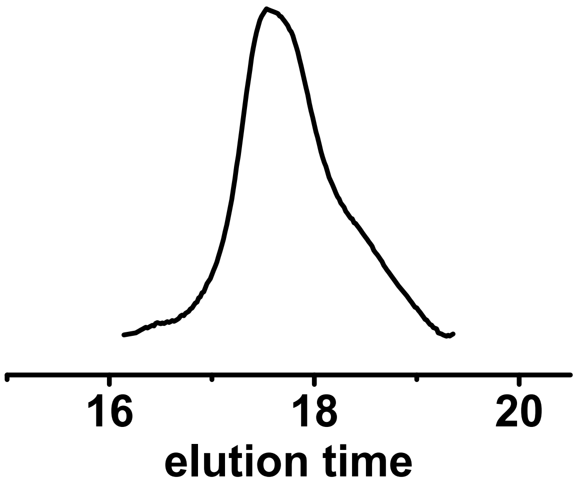

Figure S2. GPC trace of Rif-Br_3 macroinitiator.

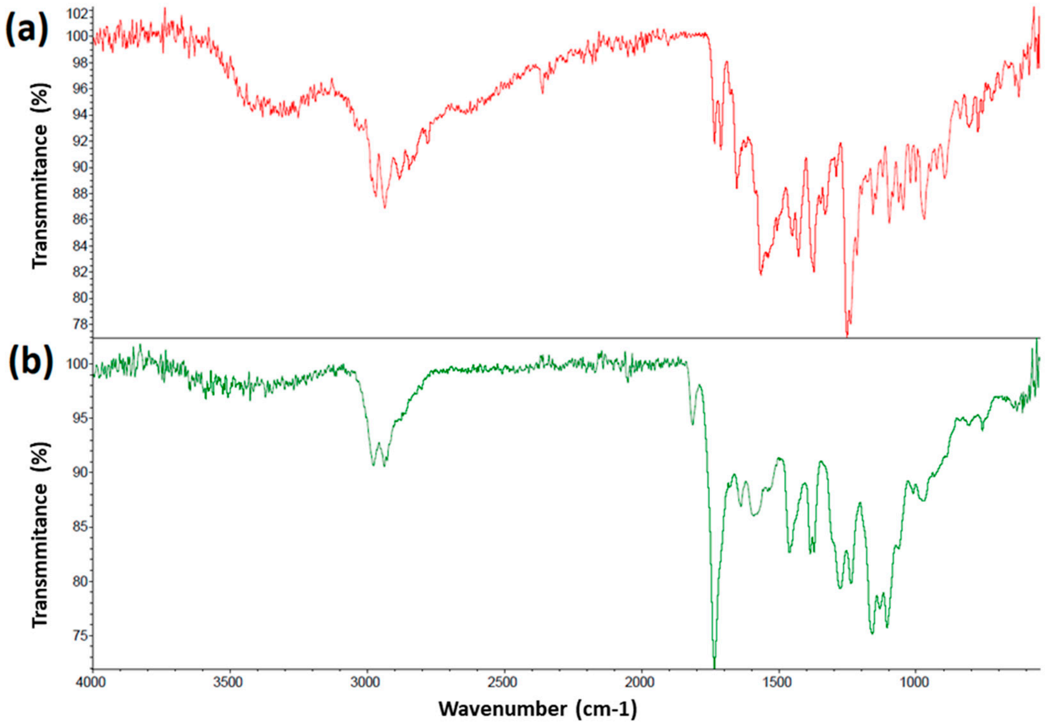

Figure S3. FT-IR characterization of (**a**) rifampicin and (**b**) Rif-Br_3 macroinitiator.

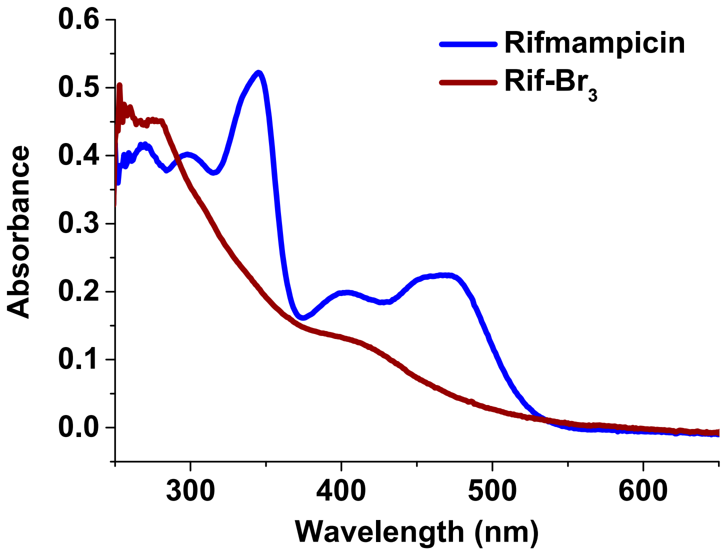

Figure S4. UV-vis spectrum of rifampicin and Rif-Br_3 macroinitiator in THF.

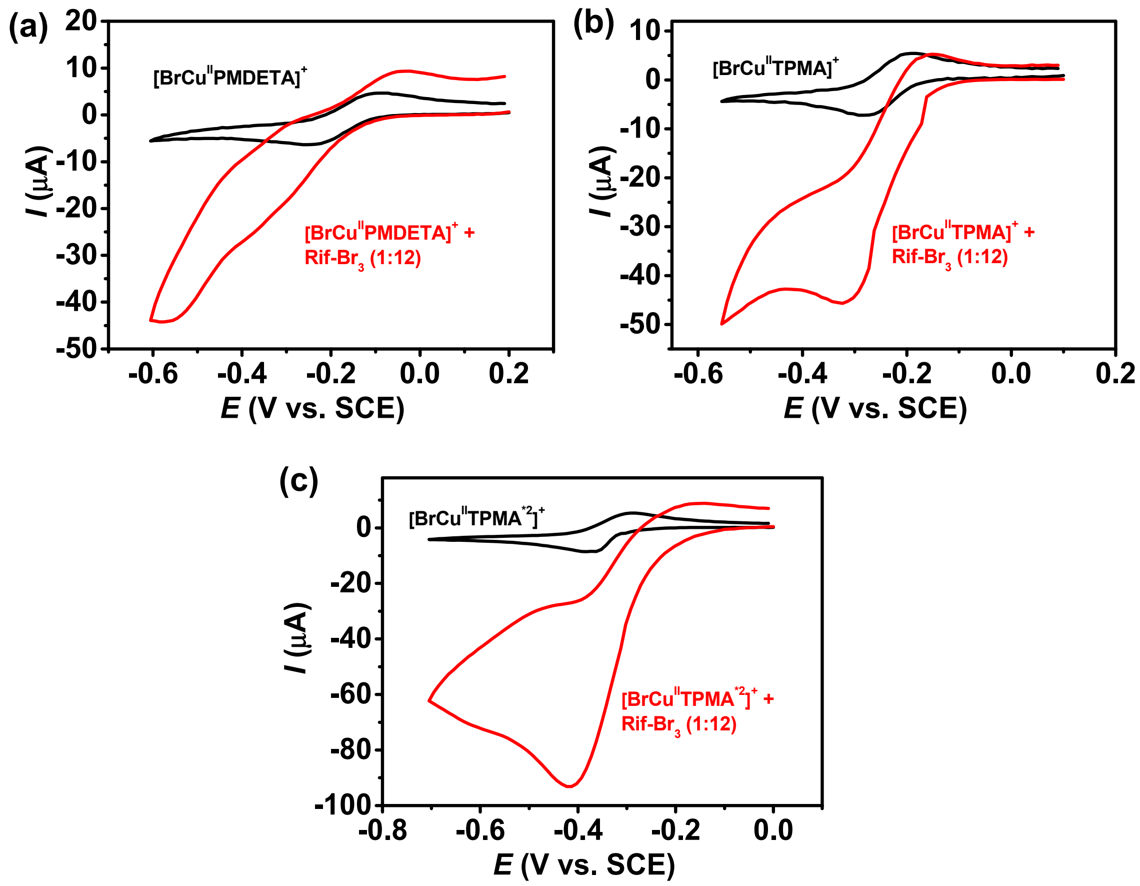

Figure S5. Cyclic voltammogram of 0.8 mM $Cu^{II}Br_2$/L in DMF containing 0.2 M TBAP in the absence (black line) and in the presence of 9.8 mM Rif-Br_3 (red line) recorded at v = 0.1 V·s^{-1}, where L (ligand) is (**a**) PMDETA, (**b**) TPMA and (**c**) TPMA^{*2}.

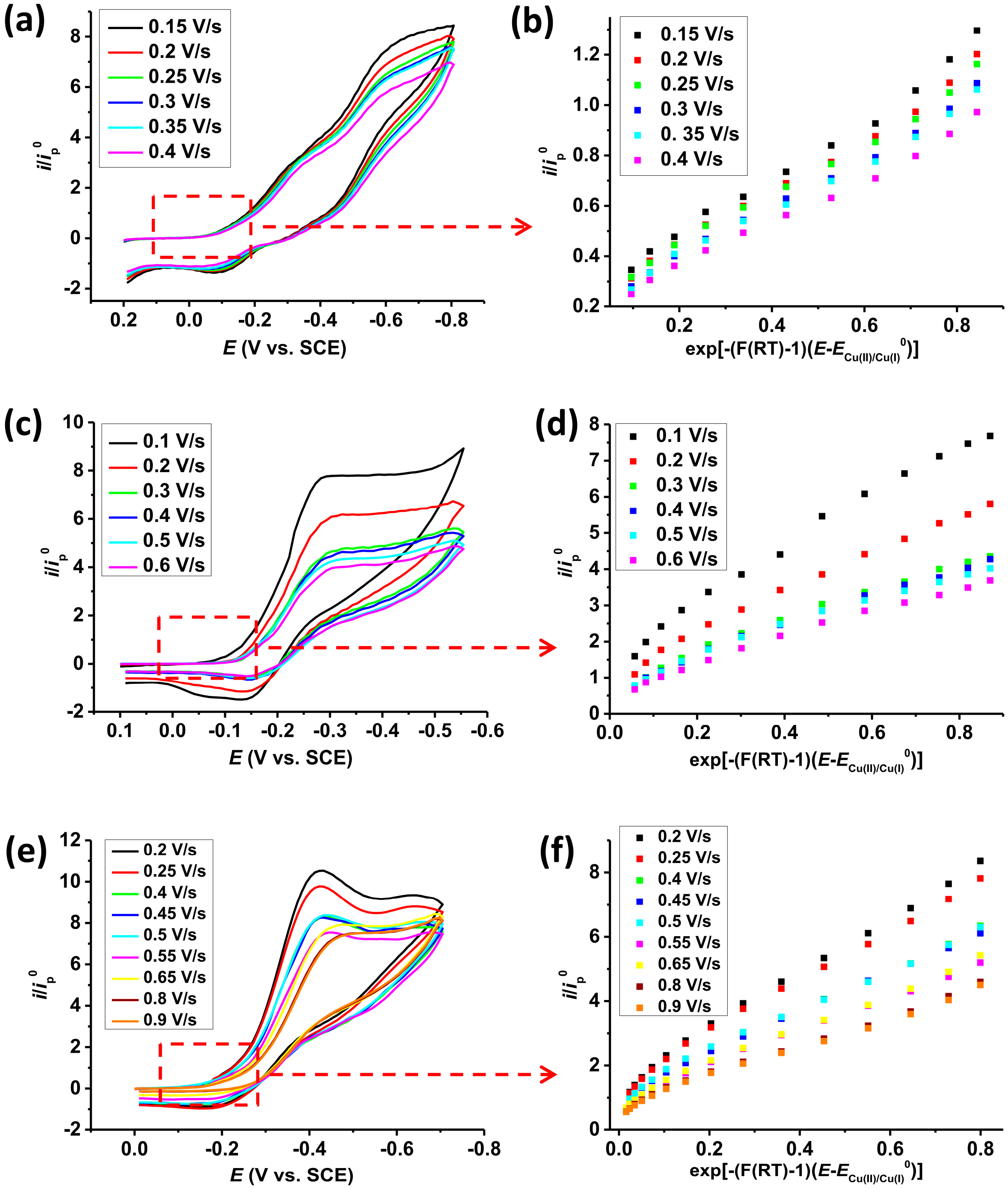

Figure S6. Cyclic voltammograms of 0.8 mM $Cu^{II}Br_2/L$ in DMF recorded at a different scan rates (given next to the curves) in the presence of 9.8 mM Rif-Br_3 (3 Br molecules) and 39.9 mM TEMPO; the current was normalized with respect to the peak current (i_p^0) recorded in the absence of Rif-Br_3, where L (ligand) is (**a**) PMDETA, (**c**) TPMA and (**e**) TPMA^{*2}; Foot-of-the-wave analysis of the catalytic peak to determine k_a, the slope a of the plots of $i/i_p°$ vs. exp[$-F(E-E_{Cu(II)/Cu(I)}^0)/RT$]: $a = 2.24\sqrt{\frac{k_a C_A^0 RT}{Fv}}$, where i—catalytic current, i_p^0—reversible one-electron reduction of the copper catalyst complex in the absence of Rif-Br_3, C_A^0—initial Rif-(PnBA-Br)$_3$ concentration, F—Faraday constant, R—gas constant, T = 298 K, $E^0_{Cu^{II}/Cu^{I}}$ is the half wave potential of the $Cu^{II}Br_2/L$, using (**b**) PMDETA, (**d**) TPMA and (**e**) TPMA^{*2} as a ligand.

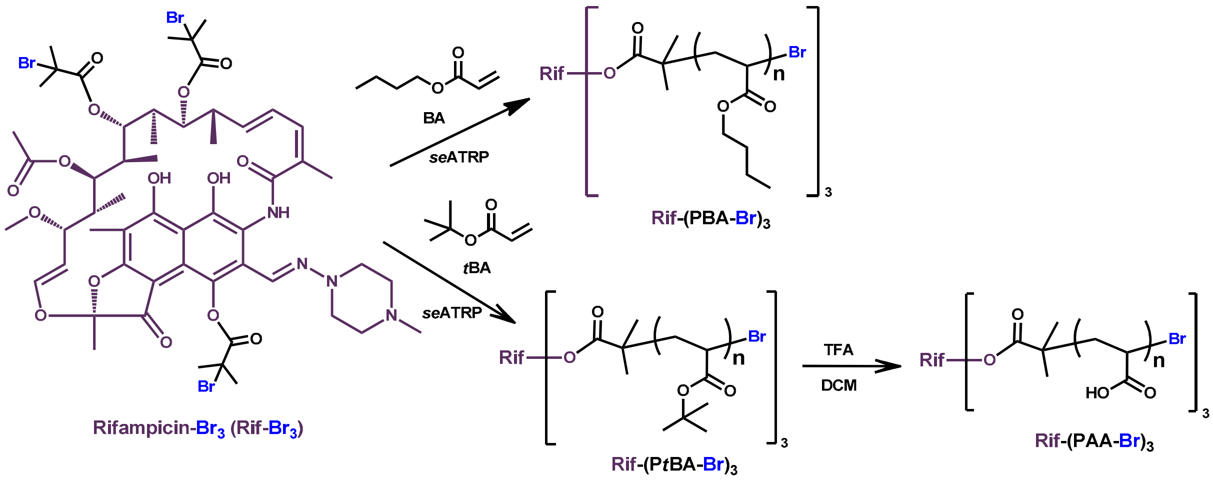

Figure S7. Synthetic route for the preparation of rifampicin-based macromolecules with acrylates (P*n*BA and P*t*BA) and poly(acrylic acid) (PAA) side chains.

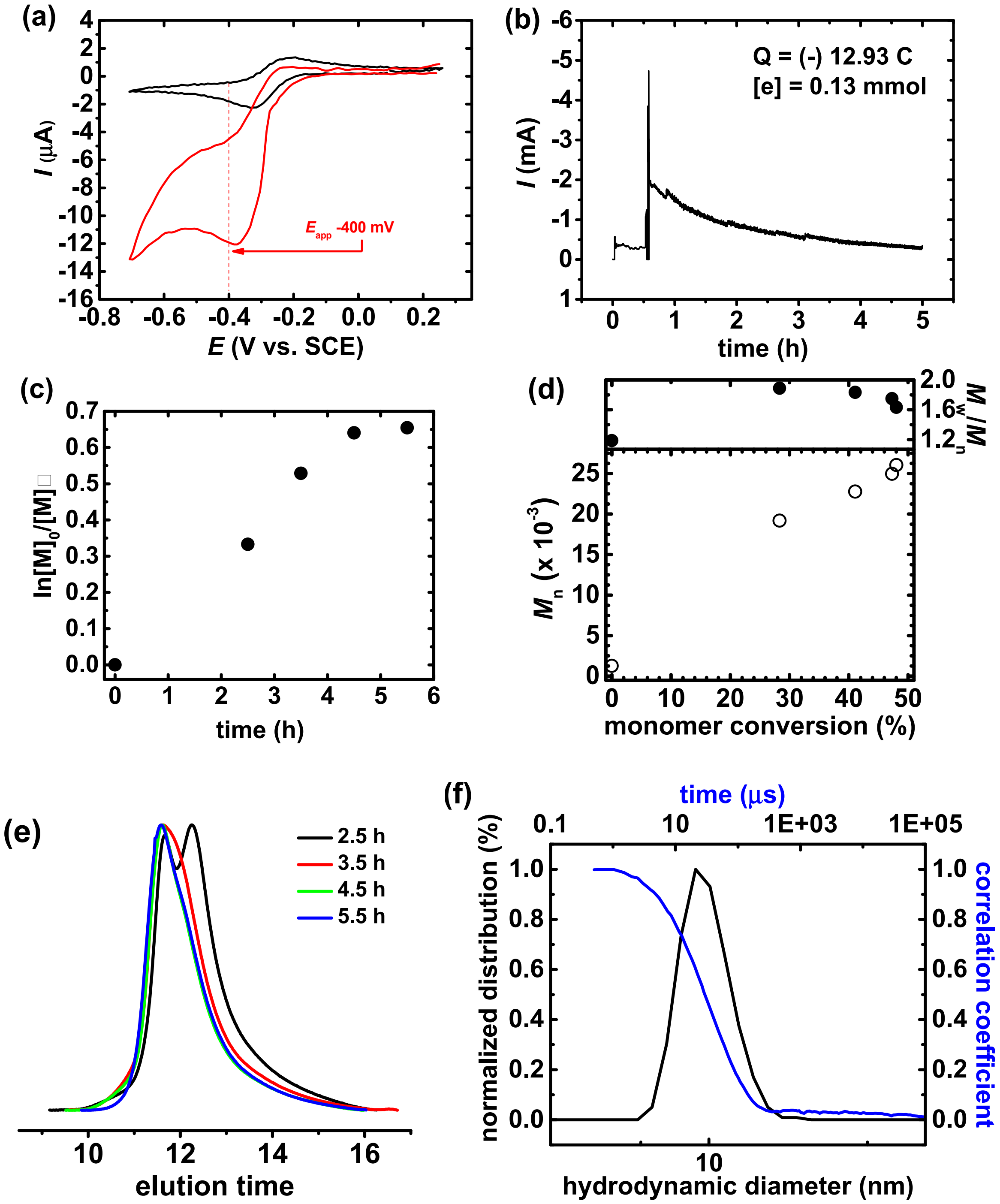

Figure S8. (**a**) Cyclic voltammogram of 0.44 mM $Cu^{II}Br_2$/TPMA*2 in 15% (*v*/*v*) *n*BA/DMF ($[nBA]_0$ = 1.10 M) containing 0.2 M TBAP in the absence (black line) and in the presence of 1.35 mM Rif-Br_3 (red line) recorded at v = 0.1 V·s^{-1}, (**b**) current profile vs. time for the polymerization of *n*BA from Rif-Br_3, (**c**) First-order kinetic plot of monomer conversion vs. time, (**d**) M_n and M_w/M_n vs. monomer conversion, (**e**) GPC traces of *n*BA polymerization and their evolution over reaction time, (**f**) DLS hydrodynamic size distributions by volume of Rif-(P*n*BA-Br)$_3$. Table 1, entry 1.

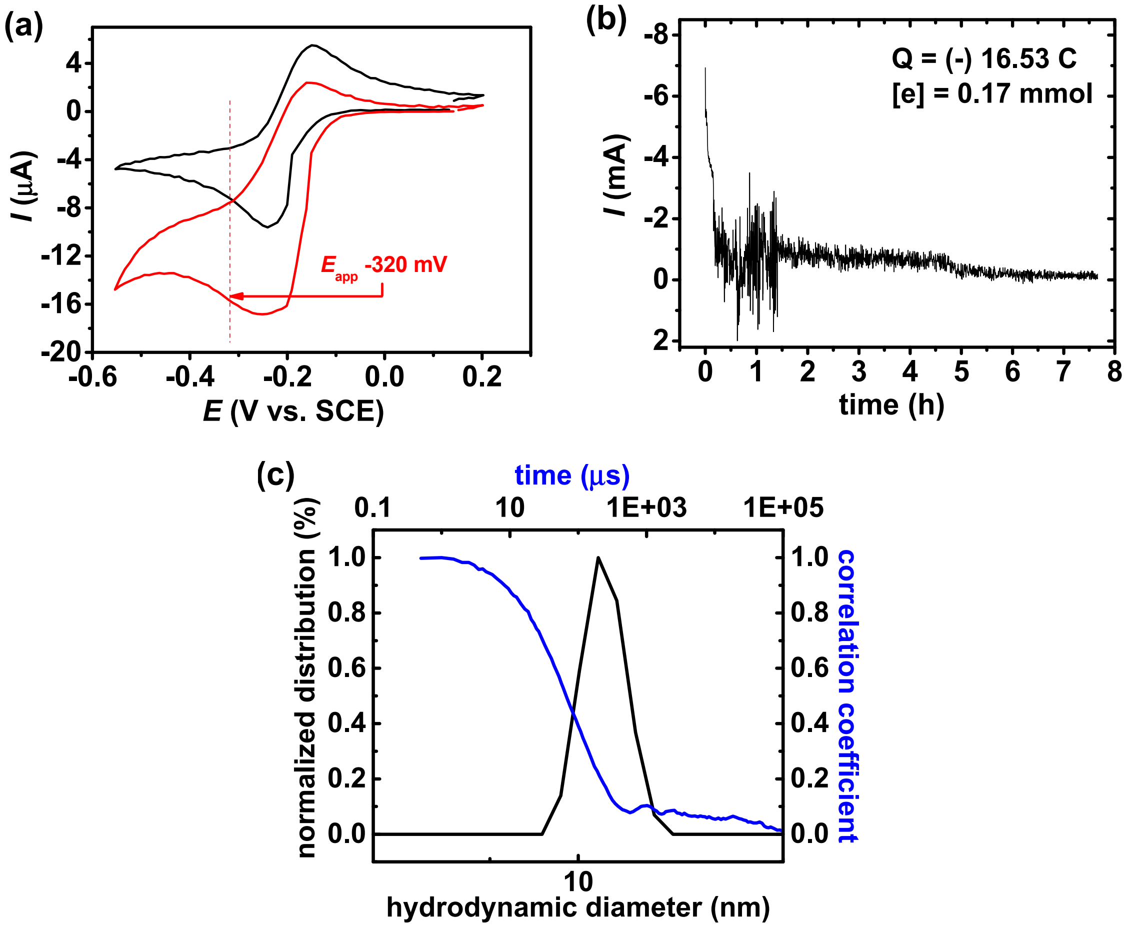

Figure S9. (**a**) Cyclic voltammogram of 0.88 mM $Cu^{II}Br_2$/TPMA in 30% (*v*/*v*) *n*BA/DMF ($[n\text{BA}]_0$ = 2.19 M) containing 0.2 M TBAP in the absence (black line) and in the presence of 2.71 mM Rif-Br_3 (red line) recorded at v = 0.1 V·s^{-1}, (**b**) current profile vs. time for the polymerization of *n*BA from Rif-Br_3, (**c**) DLS hydrodynamic size distributions by volume of Rif-(P*n*BA-Br)$_3$. Table 1, entry 2.

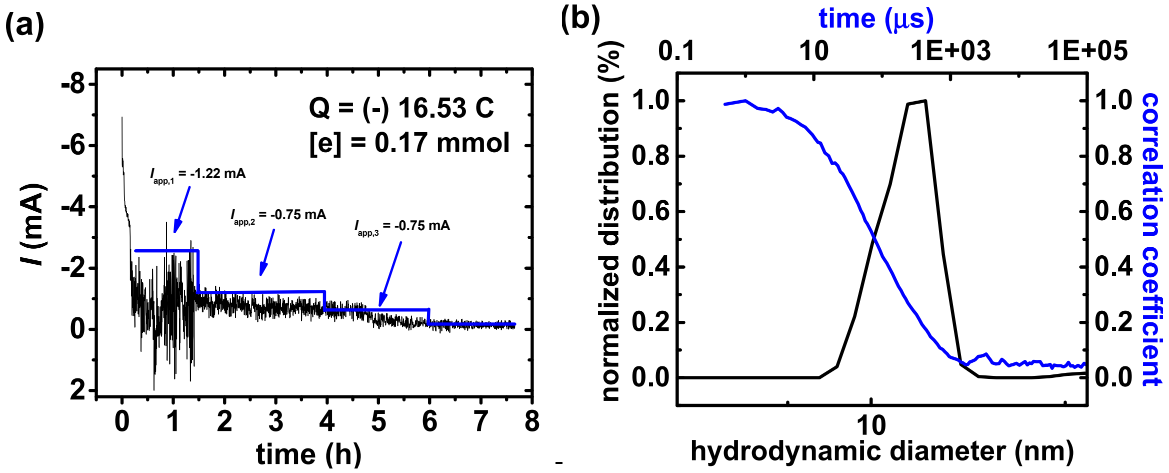

Figure S10. (**a**) Current profile vs. time for the polymerization of *n*BA from Rif-Br_3 under constant potential conditions and the determined current steps for constant current electrolysis (**b**) DLS hydrodynamic size distributions by volume of Rif-(P*n*BA-Br)$_3$. Table 1, entry 3.

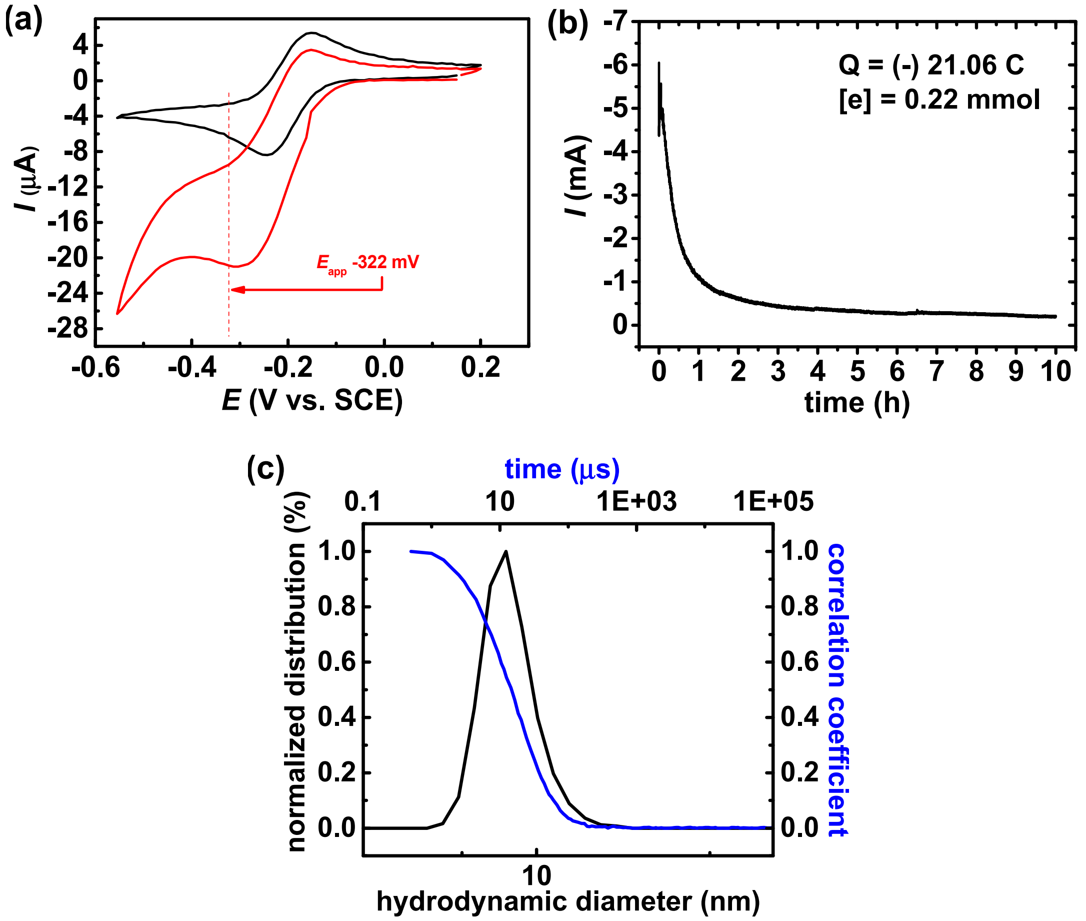

Figure S11. (**a**) Cyclic voltammogram of 0.82 mM $Cu^{II}Br_2$/TPMA in 30% (v/v) *t*BA/DMF ($[tBA]_0$ = 2.05 M) containing 0.2 M TBAP in the absence (black line) and in the presence of 6.74 mM Rif-Br_3 (red line) recorded at v = 0.1 V·s^{-1}, (**b**) current profile vs. time for the polymerization of *t*BA from Rif-Br_3, (**c**) DLS hydrodynamic size distributions by volume of Rif-(P*t*BA-Br)$_3$. Table 1, entry 4.

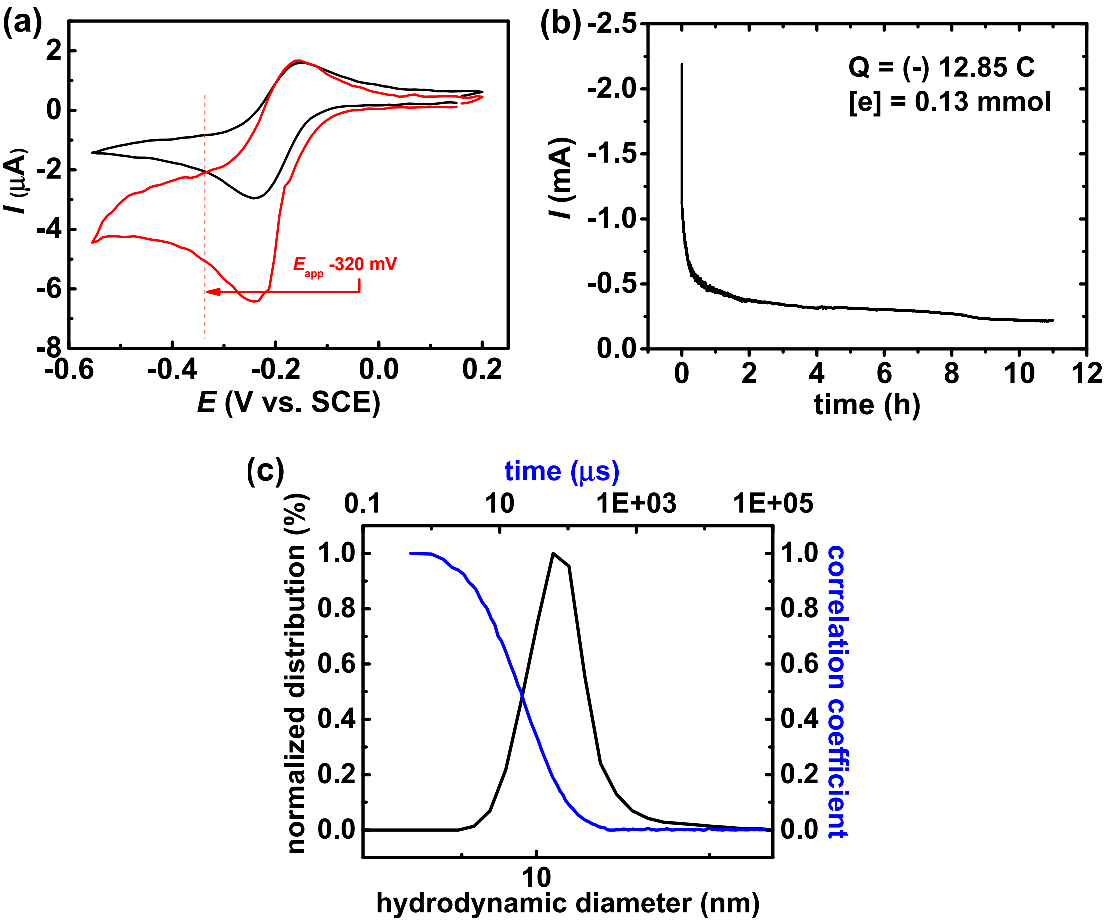

Figure S12. (**a**) Cyclic voltammogram of 0.43 mM $Cu^{II}Br_2$/TPMA in 16% (v/v) *t*BA/DMF ($[tBA]_0$ = 1.07 M) containing 0.2 M TBAP in the absence (black line) and in the presence of 1.95 mM Rif-(P*t*BA-Br)$_3$ (red line) recorded at v = 0.1 V·s^{-1}, (**b**) current profile vs. time for the polymerization of *t*BA from Rif-(P*t*BA-Br)$_3$, (**c**) DLS hydrodynamic size distributions by volume of Rif-(P*t*BA-*b*-P*t*BA-Br)$_3$. Table 1, entry 5.

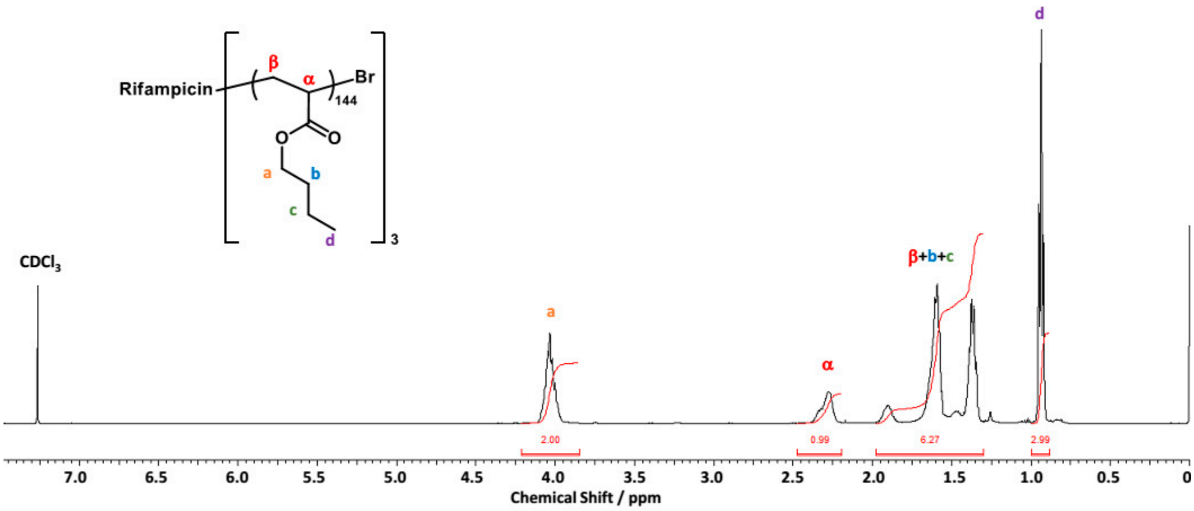

Figure S13. ^{1}H NMR spectrum of Rif-(P*n*BA-Br)$_3$ polymers (M_n = 56100, $Đ$ = 1.59) after purification (in $CDCl_3$). Table 1, entry 3.

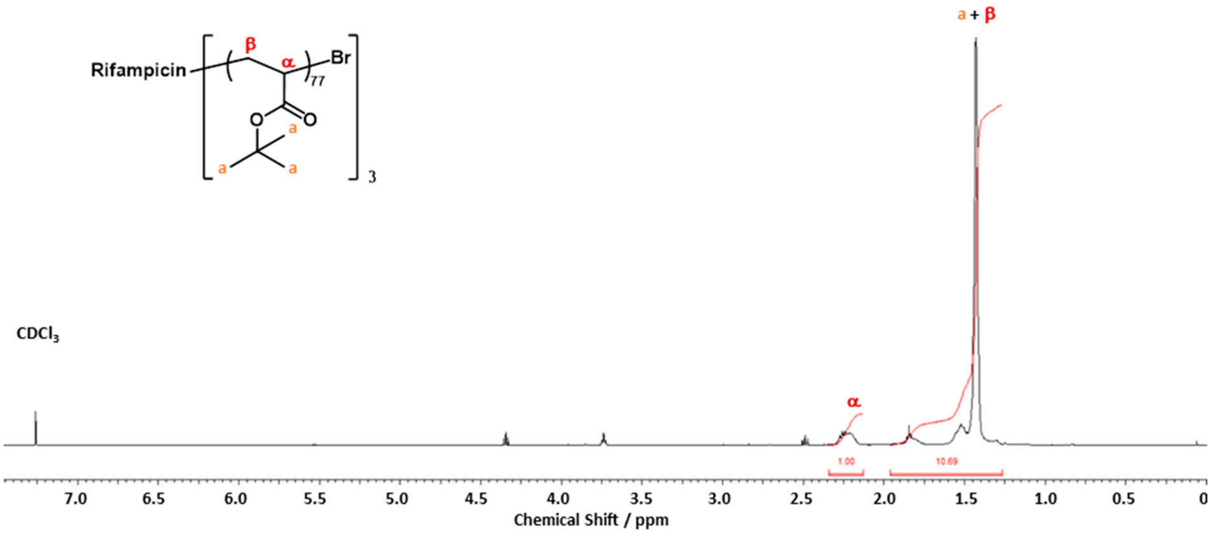

Figure S14. ^{1}H NMR spectrum of Rif-(P*t*BA-Br)$_3$ polymers (M_n = 30100, $Đ$ = 1.71) after purification (in $CDCl_3$). Table 1, entry 4.

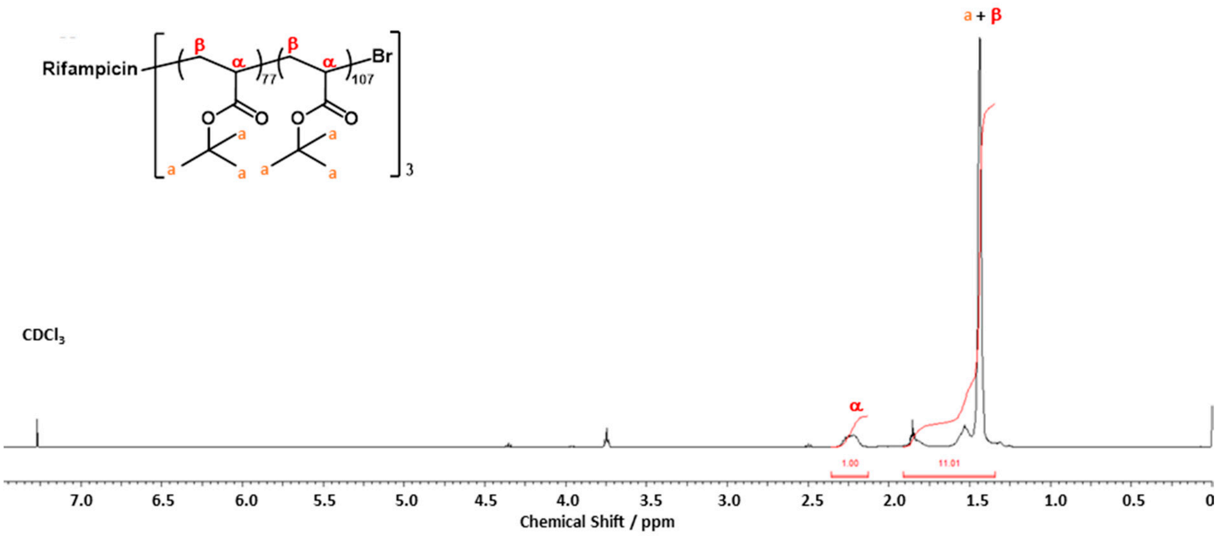

Figure S15. ^{1}H NMR spectrum of Rif-(P*t*BA-*b*-P*t*BA-Br)$_3$ polymers (M_n = 72100, $Đ$ = 1.58) after purification (in $CDCl_3$). Table 1, entry 5.

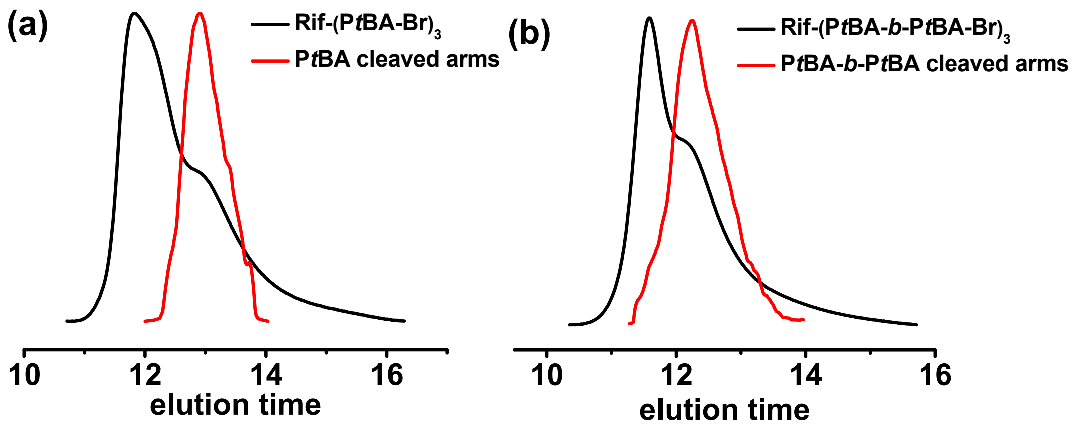

Figure S16. GPC traces of (**a**) Rif-(P*t*BA-Br)$_3$ (Table 1, entry 4) and the corresponding cleaved P*t*BA arms, and (**b**) Rif-(P*t*BA-*b*-P*t*BA-Br)$_3$ (Table 1, entry 5) and the corresponding cleaved P*t*BA-*b*-P*t*BA arms.

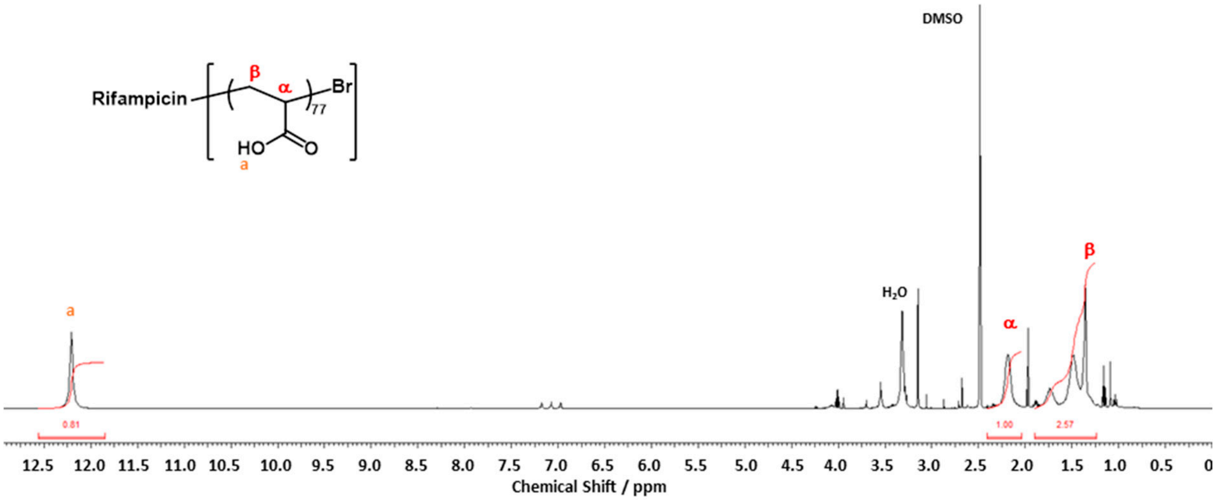

Figure S17. ^{1}H NMR spectrum of Rif-(PAA-Br)$_3$ polymers after purification (in DMSO-d_6). Table 1, entry 4.

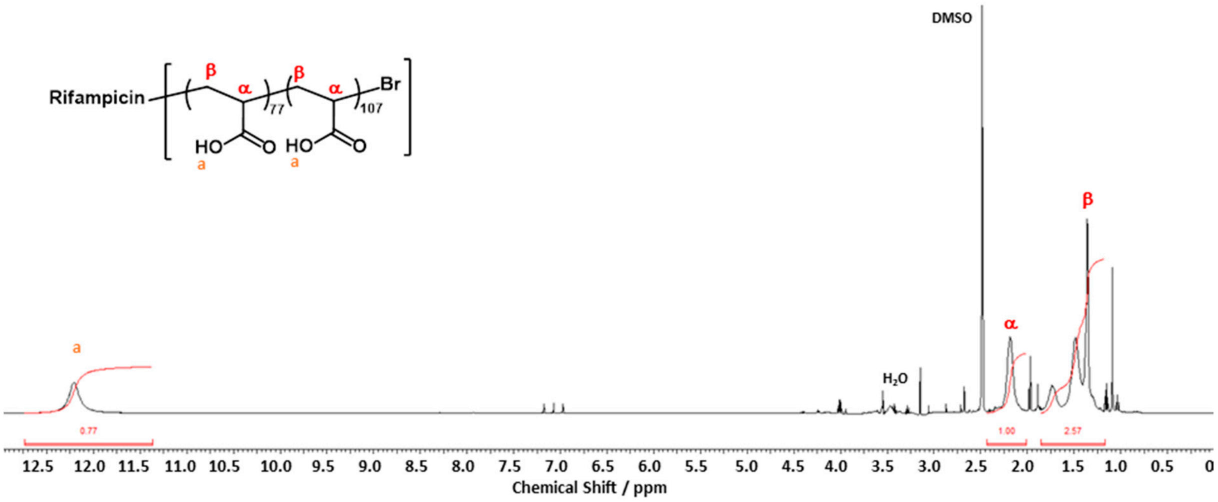

Figure S18. ^{1}H NMR spectrum of Rif-(PAA-*b*-PAA-Br)$_3$ polymers after purification (in DMSO-d_6). Table 1, entry 5.

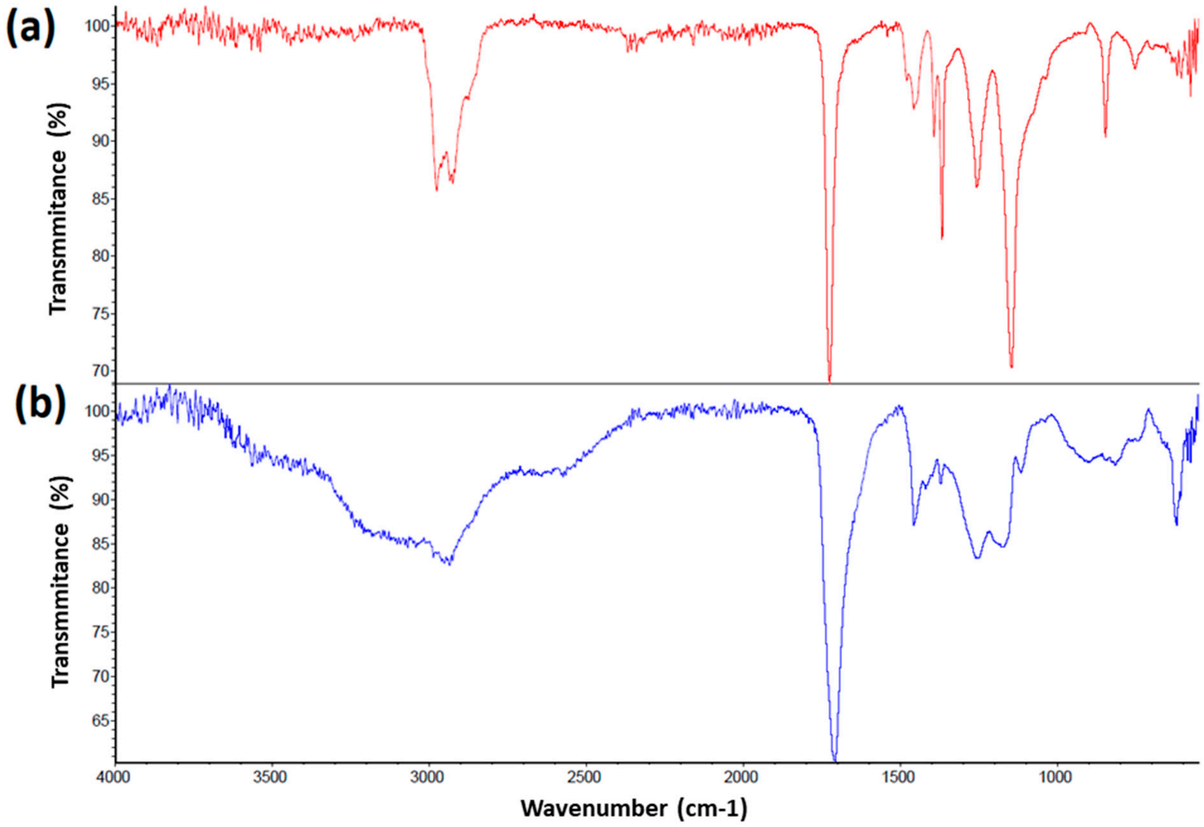

Figure S19. FT-IR characterization of (**a**) Rif-(P*t*BA-Br)3 (Table 1, entry 4) and (**b**) Rif-(PAA-Br)3.

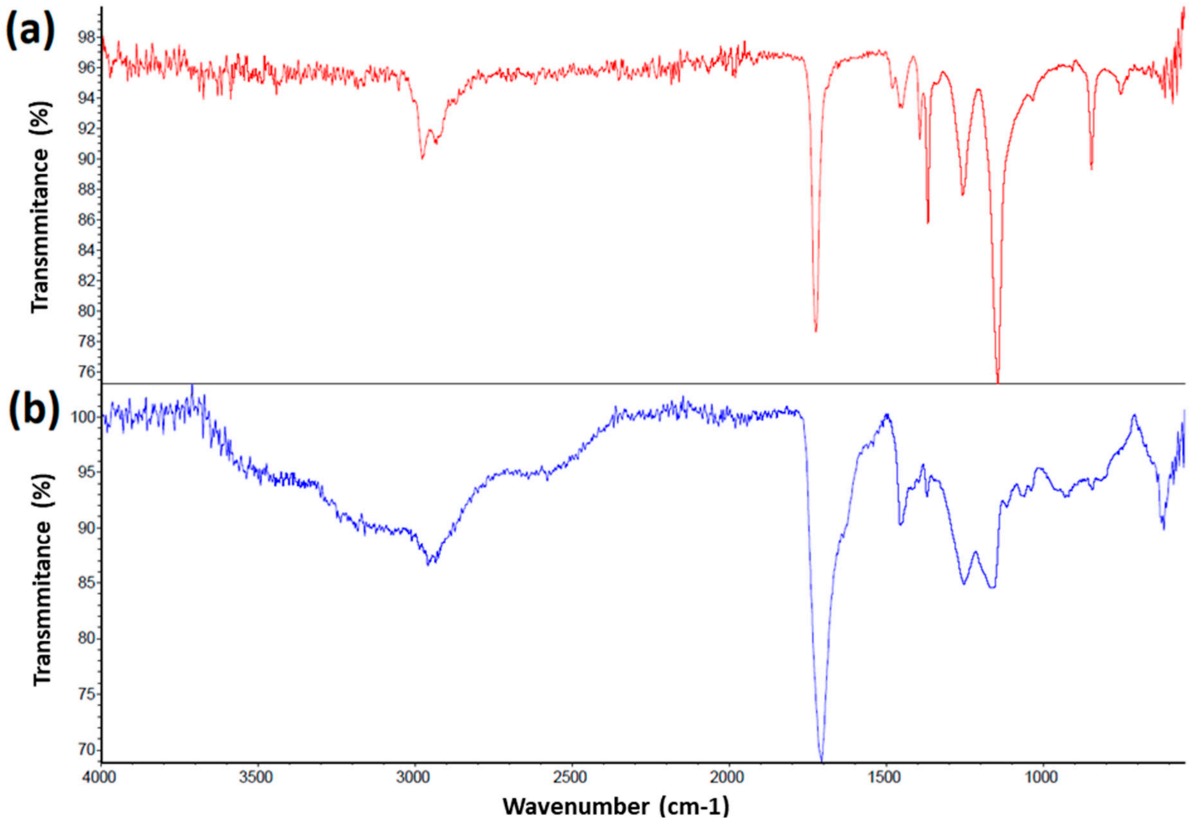

Figure S20. FT-IR characterization of (**a**) Rif-(P*t*BA-*b*-P*t*BA-Br)3 (Table 1, entry 5) and (**b**) Rif-(PAA-*b*-PAA-Br)3.

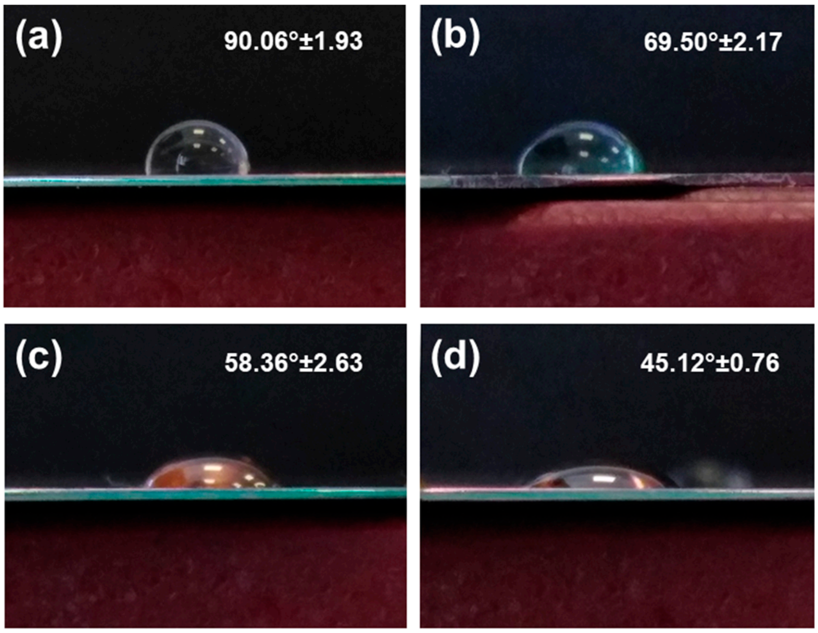

Figure S21. Water contact angle images of (a) Rif-(P*t*BA-Br)$_3$ (Table 1, entry 4) and (b) corresponding Rif-(PAA-Br)$_3$, and diiodomethane contact angle images of (c) Rif-(P*t*BA-Br)$_3$ (Table 1, entry 4) and (d) corresponding Rif-(PAA-Br)$_3$.

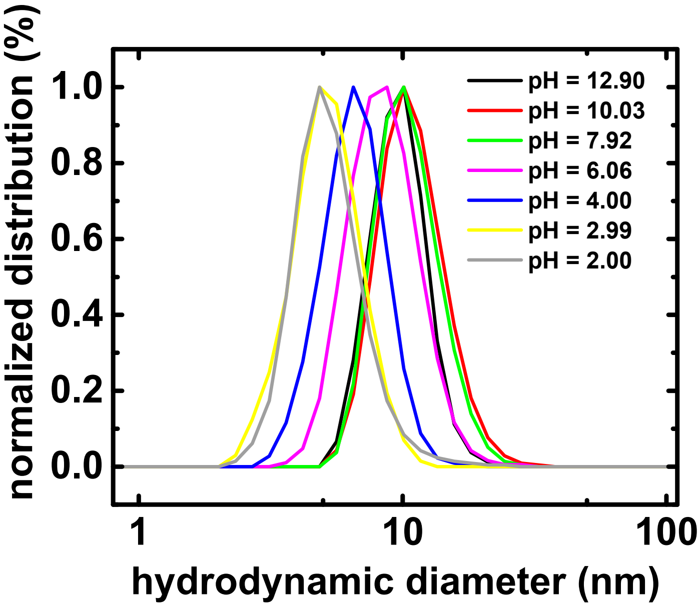

Figure S22. DLS hydrodynamic size distributions by volume of Rif-(P*t*BA-*b*-P*t*BA-Br)$_3$ in different pH.

Table S1. Calculation of Cu^I/Cu^{II} ratio for the preparation of rifampicin-based macromolecules.

Entry (according to Table 1)	k_p^{app} (h^{-1}) [a]	[$P_n^•$] (M × 10^{10}) [a]	K_{ATRP} (× 10^8) [b]	[P_n-Br] (mM)	[Cu^I]/[Cu^{II}] [c]	[Cu^IL^+] (%)	[Br–$Cu^{II}L^+$] (%)
1	0.133	13.3	0.0047	1.35	0.02	2.1	97.9
2	0.109	10.9	1.30	2.71	31.06	96.9	3.1
3	0.095	9.53	1.30	2.71	27.07	96.4	3.6
4	0.137	13.3	1.30	6.74	15.18	93.8	6.2
5	0.097	9.42	1.30	1.95	37.17	97.4	2.6

[a] The radical concentration $[P_n^•]$ was calculated according to the equation defined as $[P_n^•] = \left(\frac{d\ln[M]}{dt}\right)(k_p)^{-1}$ [1], where $\frac{d\ln[M]}{dt}$ values were calculated from the first order kinetics plots (S8c in SI, 1a, 2a, 3a and 4a) [2], $k_p = 2.77 \times 10^4$ $M^{-1}s^{-1}$ for *n*BA polymerization [3], $k_p = 2.86 \times 10^4$ $M^{-1}s^{-1}$ for *t*BA polymerization [4], [b] entry 1: K_{ATRP} = 4.7×10^{-5} was determined theoretically for the CuI/TPMA$^+$ catalyst acetonitrile at 25°C [5]; entry 2–5: K_{ATRP} = 1.3×10^{-8} was determined for the Cu^I/TPMA$^+$ catalyst in methyl acrylate/acetonitrile 50/50 (v/v) at 50°C [6] [c] The Cu^I/Cu^{II} ratio was calculated according to the equation defined as $\frac{[Cu^ITPMA^+]}{[Br-Cu^{II}TPMA^+]} = \frac{[P_n^•]}{[P_n-Br]K_{ATRP}}$ [6].

Table S2. Theoretical Al^{3+} concentration in solution and polymer by monomer conversion.

Entry (according to Table 1 and S1)	Q [a] (C)	$n_{Al^{3+}}$ [b] (mol × 10^5)	[Al^{3+}]$_{solution}$ [c] (ppm by wt)	[Al^{3+}]$_{polymer}$ [d] (ppm by wt)
1	12.93	4.47	48.9	11.7
2	16.53	5.71	98.6	25.1
3	16.53	5.71	98.6	26.3
4	21.06	7.28	128.0	48.9
5	12.85	4.44	87.8	25.8

[a] The total passed charge was calculated by integration of the chronoamperometry (CA) area ($Q = I{\cdot}t$); [b] theoretical amount of Al^{3+} in the reaction mixture was calculated from CA: $n_{Al^{3+}} = Q/F/3$ where F = 96485 C/mol; [c] the Al concentration in the reaction mixture was calculated according to the equation defined as: $[Al^{3+}]_{solution} = [Al^{3+}]\ MW_{Al}\ /\ wt_{total} \times 1000000$ where solution density was assumed as $(d) = d_{DMF} \cdot \%\ (v/v)_{DMF} + d_{monomer} \cdot \%\ (v/v)_{monomer}$; [d] the Al concentration in pure polymer sample was determined as follows: $[Al^{3+}]_{polymer} = [Al^{3+}]_{solution}\ /\ df \cdot$ conversion, where df is dilute factor, df = 2 [7].

Table S3. Calculation of theoretical Dead Chain Fraction (DCF_{theo}) for polymerization of acrylates at low copper catalyst loading.

Entry (according to Table 1)	[$P_n^•$] [a] (M × 10^{10})	[D] [b] (M × 10^6)	[P_n–Br] (mM)	DCF_{theo} [c] (%)
1	13.3	3.52	1.35	0.26
2	10.9	3.30	2.71	0.12
3	9.53	2.51	2.71	0.09
4	13.3	6.37	6.74	0.09
5	9.42	3.52	1.95	0.18

[a] The radical concentration $[P^•]$ was calculated according to the equation defined as $[P_n^•] = \left(\frac{d\ln[M]}{dt}\right)(k_p)^{-1}$ [1], where $\frac{d\ln[M]}{dt}$ values were calculated from the first order kinetics plots (Figure 2a) [8], entry 1: $k_p = 2.86 \times 10^4$ $M^{-1}s^{-1}$ [4], entry 2-5: $k_p = 2.77 \times 10^4$ $M^{-1}s^{-1}$ [9]. [b] The concentration of terminated chains [D] was calculated according to the equation defined as $[D] = k_t[P]^2t$ where t (denote reaction time) = 19800 s (entry 1), t = 27601 s (entry 2 and 3), t = 36000 s (entry 4) and t = 39600 s (entry 5), $k_t = 1.0 \times 10^8$ $M^{-1}s^{-1}$ [10]. [c] $DCF = \left(\frac{[D]}{[P-X]_0}\right) \times 100\%$ [1].

Table S4. Results of the detaching of polymer arms from rifampicin-based macromolecules.

Entry (according to Table 1)	$M_{n,theo}$ (×10^{-3}) [a] (chain)	$DP_{n,theo}$ [b] (chain)	$M_{n,app}$ (×10^{-3}) [c] (chain)	$DP_{n,app}$ [b] (chain)	M_w/M_n [c]	f_i [d] (%)
4	9.9	77	23.4	182	1.20	42
5	23.6	184	44.6	348	1.42	53

[a] $M_{n,th}$ = ([tBA]$_0$/[Rif-Br$_3$]$_0$) × conversion × M_{tBA}, [tBA]$_0$—initial monomer concentration, [Rif-Br$_3$]$_0$—initiator concentration; [b] established according to Table 1; [c] apparent M_n and M_w/M_n of the arms cleaved from the rifampicin-based macromolecules determined by THF GPC (PS standards); [d] efficiency of initiation: f_i = ($DP_{n,theo}$ (per chain)/$DP_{n,app}$ (per chain)) × 100%.

Table S5. Experimental values of contact angles, parameters of free surface energy (FSE) as calculated by Owens-Wendt method for rifampicin-based polymer coatings.

Entry (according to Table 1)	Polymer	Experimental values of θ (°)				Parameters of FSE (mJ/m^2) water-diiodomethane		
		Diiodomethane	Standard deviation	Water	Standard deviation	γ_S	γ_S^d	γ_S^p
4	PtBA	58.36	2.63	90.06	1.93	29.82	26.94	2.88
	PAA	45.12	0.76	69.50	2.17	41.09	30.32	10.77
5	PtBA	70.64	1.30	92.34	0.96	23.54	19.55	3.99
	PAA	50.49	0.91	81.96	1.75	35.01	30.06	4.95

Table S6. Volume mean diameter of rifampicin-based macromolecules at varying pH.[a]

Sample	pH	Hydrodynamic diameter (nm)
1	12.90	14.59 ± 0.60
2	10.03	11.96 ± 0.80
3	7.92	11.48 ± 1.16
4	6.06	9.21 ± 1.35
5	4.00	6.96 ± 0.50
6	2.99	5.48 ± 0.84
7	2.00	5.70 ± 0.29

[a] The experiment was conducted for the polymer sample received according to Table 1, entry 5 after acidic hydrolysis.

References

1. Zhong, M.; Matyjaszewski, K. How fast can a CRP be conducted with preserved chain end functionality? *Macromolecules* **2011**, *44*, 2668–2677, doi:10.1021/ma102834s.
2. Chmielarz, P.; Krys, P.; Wang, Z.; Wang, Y.; Matyjaszewski, K. Synthesis of well-defined polymer brushes from silicon wafers *via* surface-initiated *se*ATRP. *Macromol. Chem. Phys.* **2017**, *218*, 1700106, doi:10.1002/macp.201700106.
3. Buback, M.; Kurz, C.H.; Schmaltz, C. Pressure dependence of propagation rate coefficients in free-radical homopolymerizations of methyl acrylate and dodecyl acrylate. *Macromol. Chem. Phys.* **1998**, *199*, 1721–1727, doi:10.1002/(SICI)1521-3935(19980801)199:8<1721::AID-MACP1721>3.0.CO;2-5.
4. Dervaux, B.; Junkers, T.; Schneider-Baumann, M.; Du Prez, F.E.; Barner-Kowollik, C. Propagation rate coefficients of isobornyl acrylate, *tert*-butyl acrylate and 1-ethoxyethyl acrylate: A high frequency PLP-SEC study. *J. Polym. Sci. A Polym. Chem.* **2009**, *47*, 6641–6654, doi:10.1002/pola.23706.
5. Bortolamei, N.; Isse, A.A.; Di Marco, V.B.; Gennaro, A.; Matyjaszewski, K. Thermodynamic properties of copper complexes used as catalysts in atom transfer radical polymerization. *Macromolecules* **2010**, *43*, 9257–9267, doi:10.1021/ma101979p.

6. Wang, Y.; Kwak, Y.; Buback, J.; Buback, M.; Matyjaszewski, K. Determination of ATRP equilibrium constants under polymerization conditions. *ACS Macro Lett.* **2012**, *1*, 1367–1370, doi:10.1021/mz3005378.
7. Chmielarz, P. Synthesis of α-D-glucose-based star polymers through simplified electrochemically mediated ATRP. *Polymer* **2016**, *102*, 192–198, doi: 10.1016/j.polymer.2016.09.007.
8. Chmielarz, P.; Fantin, M.; Park, S.; Isse, A.A.; Gennaro, A.; Magenau, A.J.D.; Sobkowiak, A.; Matyjaszewski, K. Electrochemically mediated atom transfer radical polymerization (*e*ATRP). *Prog. Polym. Sci.* **2017**, *69*, 47–78, doi:10.1016/j.progpolymsci.2017.02.005.
9. Buback, M.; Kurz, C.H.; Schmaltz, C. Pressure dependence of propagation rate coefficients in free-radical homopolymerizations of methyl acrylate and dodecyl acrylate. *Macromolecular Chemistry and Physics* **1998**, *199*, 1721–1727, doi:10.1002/(SICI)1521-3935(19980801)199:8<1721::AID-MACP1721>3.0.CO;2-5.
10. Barth, J.; Buback, M.; Hesse, P.; Sergeeva, T. Termination and transfer kinetics of butyl acrylate radical polymerization studied via SP-PLP-EPR. *Macromolecules* **2010**, *43*, 4023–4031, doi:10.1021/ma1006039.

© 2020 by the authors. Submitted for possible open access publication under the terms and conditions of the Creative Commons Attribution (CC BY) license (http://creativecommons.org/licenses/by/4.0/).
